# Screening and mechanistic evaluation of antioxidants for mitigating beany flavor formation during pea protein isolation

**DOI:** 10.1016/j.crfs.2026.101400

**Published:** 2026-04-09

**Authors:** José Villacís-Chiriboga, Elahe Sharifi, Madeleine Jönsson, Karin Wendin, Mehdi Abdollahi

**Affiliations:** a Department of Life Sciences - Food and Nutrition Science, Chalmers University of Technology, Gothenburg, SE-41296, Sweden; b Department of Food and Meal Science, Kristianstad University, Kristianstad, SE-291 39, Sweden; c Department of Food Science, University of Copenhagen, Frederiksberg C, DK-1958, Denmark

**Keywords:** Plant protein, Antioxidants, lipid oxidation, LOX, Off-flavor, Sensory

## Abstract

This study investigated the role of lipid oxidation in off-flavor development during pea protein (PP) extraction via alkaline solubilization and isoelectric precipitation and its mitigation through antioxidant treatment. Four antioxidants (coffee extract, caffeic acid, catechins, and Duralox®) were screened by tracking oxidation markers: malondialdehyde (MDA), hydroxyoctadecadienoic acids (HODE), and volatile compounds. Catechins and Duralox® effectively reduced the formation of HODE and beany volatiles in PP. Further optimization explored the dosage effect of catechins (250–2000 ppm), Duralox® (0.25–2.0%), and their combinations (25:75, 50:50, 75:25). Higher catechins concentrations nearly eliminated hexanal formation by suppressing lipoxygenase activity and free radicals generation, indicating dual enzymatic and non-enzymatic inhibition. Duralox® limited primary oxidation but increased radicals and volatiles at higher doses. Sensory analysis confirmed that catechins reduced beany off-flavors in PP but introduced bitterness and astringency. Overall, these findings highlight the potential of targeted antioxidant treatments to mitigate lipid oxidation-driven off-flavor formation during PP extraction.

## Highlights

• Lipid oxidation (LO) drives off-flavor formation during wet fractionation (WF).
• Hydroxyoctadecadienoic acids are effective biomarkers of LO during WF.
• Addition of catechins effectively inhibits beany flavor development during WF.
• Catechins suppressed both enzymatic and radical-driven lipid oxidation pathways.
• Duralox® and caffeic acid are not effective antioxidants during pea protein WF.

## Graphical abstract

**Figure:**
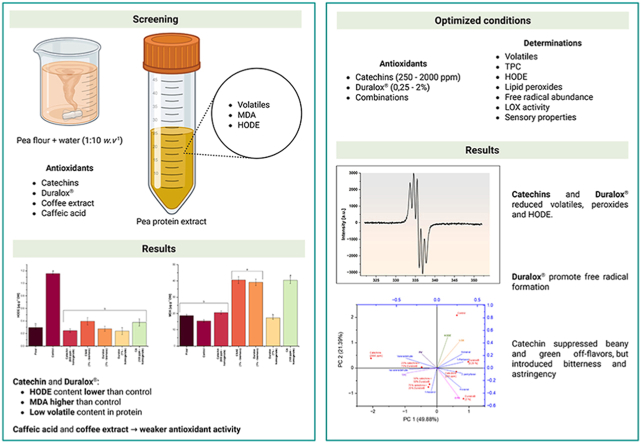

## 1 Introduction

The growing demand for plant-based proteins, fueled by ethical, environmental, and animal welfare concerns, has spotlighted pea (*Pisum sativum* L.) due to its high protein content (21 – 24% DW), adaptability to adverse weather, low allergenicity, and other favorable characteristics (Gultekin Subasi et al., 2024). Maximizing these benefits for food applications requires efficient protein extraction techniques. One commonly used approach is wet fractionation process, which involves immersing pea flour in a 10 – 15 water ratio, followed by alkaline solubilization, isoelectric precipitation and drying to yield a protein content ≥70% (Sajib et al., 2023).

However, the widespread application of pea protein, like other legume proteins, is hindered by off-flavors that reduce consumer acceptance at higher inclusion levels (Gultekin Subasi et al., 2024). These off-flavors, typically described as green, grassy, or beany, may be inherent to the pulse or formed during harvesting, processing, or storage. The main origin is the oxidation of unsaturated fatty acids (USFAs), such as linoleic and linolenic acids, yielding volatile aldehydes, ketones, and alcohols with low odor thresholds (Frank et al., 2001). Lipid oxidation can be enzymatically initiated by lipoxygenase (LOX), which catalyzes the degradation of USFAs into hydroperoxides that subsequently break down into a wide range of volatile compounds, widely recognized as major contributors to undesirable flavors. Hydroperoxides can also form through autoxidation or photooxidation of USFAs via free radical chain reactions in the presence of oxygen or light, offering alternative non-enzymatic routes for off-flavor generation in pea (Trindler et al., 2022). The degradation of USFAs through these pathways is influenced by various external factors (*e.g*. temperature, initiators, antioxidants, oxygen availability, and surface exposure to air) as well as internal factors such as LOX activity, USFA content, and degree of unsaturation. Additionally, metal ions and light can catalyze the breakdown of hydroperoxide intermediates, further promoting the formation of volatile off-flavor compounds (Wang et al., 2021) which are all present to a different extent during the wet fractionation.

Wet fractionation can significantly alter the quantity and profile of flavor compounds. For example, Murat et al. (2013) and Xu et al. (2020) reported that many water-soluble molecules are readily lost during pea protein extraction. However, new flavor-active compounds emerged, either released from protein or lipid conjugates via alkaline hydrolysis or newly formed through lipid oxidation during extraction. Recent studies have shown that off-flavor compounds can become concentrated in the protein fraction by up to 4 to 100-fold after wet fractionation (Gultekin Subasi et al., 2024; Manouel et al., 2024), presenting a technical hurdle for improving the sensory quality of pea-based protein products. Gao et al. (2020) demonstrated that the pH of the solubilization step strongly affects levels of key beany-flavor compounds such as hexanal, 1-pentanol, and 3-methyl-1-butanol, as LOX becomes activated upon water addition and its subsequent activity, as well as the chemical pathways leading to these volatiles, is modulated by pH. In contrast, Cui et al. (2020) attributed the impact of pH on flavor profile pH-dependent interactions between flavor compounds and proteins, rather than to the extraction pH itself. This background clearly shows a gap and a lack of clear understanding of the origin of off-flavor compounds evolution during protein extraction using the alkaline solubilization/isoelectric precipitation method and the underlying drivers responsible for these changes. The complexity of the process, driven by interacting factors such as protein–flavor binding, enzymatic activity, hydrolysis, and multiple lipid oxidation pathways (*i.e.*, enzymatic, non-enzymatic, and metal-catalyzed), necessitates deeper mechanistic investigation to better engineer the wet fractionation process for off-flavor mitigation, which remains an open research gap to the best of our knowledge.

Lipid oxidation is commonly monitored using peroxides and malondialdehyde (MDA) as markers (Tullberg et al., 2016), but their short life span or high reactivity with other compounds and lack of specificity for USFA oxidation in legumes limit their usefulness during protein extraction. In contrast, hydroxyoctadecadienoic acids (HODE) are a group of specific, stable oxidation products of linoleic acid and can be formed via both enzymatic and non-enzymatic pathways, providing detailed mechanistic information about the type and source of oxidative stress (Joshi et al., 2016). Tracking the formation pathways of these compounds may yield a better understanding of off-flavor development in pea protein but has not yet been used for monitoring lipid oxidation during the protein extraction, to the best of our knowledge.

At the molecular level, fatty acid oxidation begins with the formation of alkyl radicals (R•), which can rapidly react with molecular oxygen to form alkyl peroxyl radicals (ROO•). These in turn abstract hydrogen atoms from other linoleic acid molecules, perpetuating a chain reaction and forming lipid hydroperoxides (Pospíšil, 2016). The hydroperoxides may then decompose into alkoxyl radicals (RO•) and a variety of secondary products, including aldehydes, alcohols, and epoxides (Y. Yao et al., 2024). Although these radical-mediated pathways are well established, their specific contribution to off-flavor generation during wet protein extraction remains poorly understood. Electron paramagnetic resonance (EPR) spectroscopy offers a powerful, yet underutilized, tool for detecting and characterizing short-lived free radicals in real time (Amft et al., 2020). Applying EPR to this context could help elucidate the dynamics of radical formation during extraction and their link to the generation of volatile compounds, addressing a critical gap in flavor evolution research in plant-based proteins.

Considering the very high chance of lipid oxidation during the protein extraction process, one promising avenue for mitigating off-flavor formation is the incorporation of antioxidants during the process. The use of antioxidants to inhibit free radical formation (Bi et al., 2022; Soendjaja and Girard, 2024) and the enrichment of extraction media with polyphenols to reduce LOX activity (Redrejo-Rodriguez et al., 2004; Y. Y. Zhang et al., 2019) have shown promising results in various plant protein systems. However, most studies have overlooked the protein extraction process and intermediary radical species and the mechanisms driving their formation.

Given the limited understanding of the mechanisms driving off-flavor dynamics during pea protein extraction, this study aimed to: (1) investigate the role of lipid oxidation in flavor evolution during pea protein extraction; (2) evaluate the potential of selected antioxidants, at various dosages, to mitigate the formation of oxidation markers and volatile compounds during extraction; (3) elucidate the underlying mechanisms of their inhibitory action; and (4) assess the impact of this mitigation strategy on the sensory properties of pea protein. The effects of the incorporation of four different antioxidants during the pea protein extraction on the formation of hydroperoxides, HODE and selected volatile compounds were evaluated. The most effective compounds were further tested at varying concentrations and in combination to assess their influence on aldehyde levels and overall volatile compounds profiles in isolated protein. To explain the underlying mechanisms of off-flavor mitigation, their effects on free radical suppression and LOX activity were also examined. Additionally, sensory evaluation with a trained analytical panel was conducted to determine how these chemical changes translated to perceptible differences in flavor, focusing on off-notes commonly associated with pea-based products.

## 2 Materials and methods

### 2.1 Plant material and pre-processing

The yellow pea (*Pisum sativum* L. var. Ingrid) harvested in 2024 was dried to 14% moisture at 30 °C, transported to the lab, manually cleaned to separate stones, leaves, barks and other impurities, and stored in airtight Ziploc bags at −80 °C. Dehulling was carried out using a Satake TM05C(2)-1 abrasive mill (Satake, Japan), followed by air blowing to remove any residual hulls. The dehulled peas were then milled into a fine flour with a particle size of 500 μm using a Retsch ZM 200 mill (Retsch, Haan, Germany) operated at 12 000 rpm. The resulting flour was stored in sealed bags at −80 °C to preserve its integrity.

### 2.2 Antioxidant type and concentrations screening

**Fig. 1:**
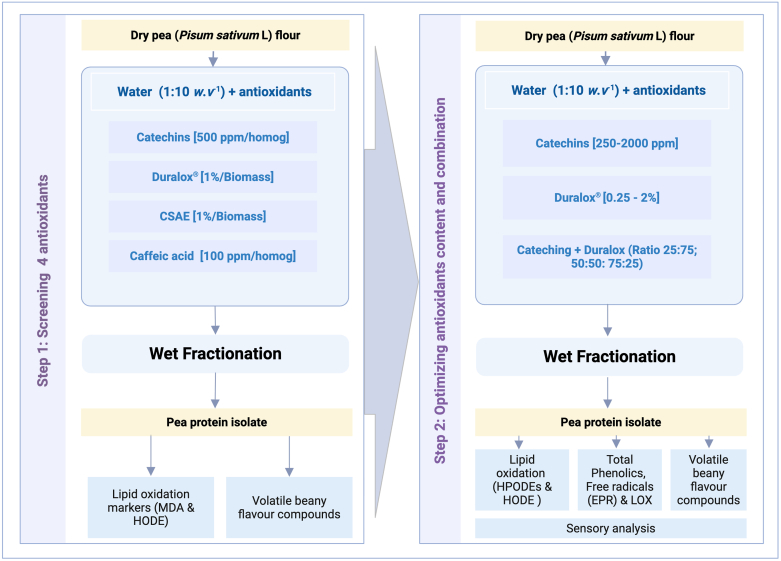
Schematic overview of the experimental set up used in this study, following a sequence of screening four antioxidants, followed by investigating the effect of the concentration and combination of the most effective ones.

Fig. 1 depicts the two-step structure of this research. To find the most promising types of antioxidants, several compounds were selected based on their structural diversity and functional properties and subjected to a screening experiment. All these antioxidants were freshly prepared before the experiments, and included catechins from green tea (Polyphenon 60, Sigma Aldrich, USA), coffee spent aqueous extract (CSAE), Duralox® oxidation management blend (a mixture of rosemary extract, ascorbic acid, tocopherols and citric acid) (Kalsec, USA), and caffeic acid (CA) (Sigma Aldrich, USA). CSAE was prepared by suspending dried ground coffee spent in water (1:10 *w*:*v*^−1^) using a magnetic stirrer for 10 min at room temperature (RT). The mixture was then centrifuged at 10 000 rpm for 10 min at 4 °C. The resulting supernatant was collected and used as an antioxidant-rich substrate.

Based on a set of pretrials, different concentrations of each antioxidant were targeted for this screening step. Catechins were added at concentrations of 500 ppm to the homogenate. CSAE and Duralox® were tested at 1.0% relative to the biomass, while Duralox® was also applied to the homogenate at concentrations of 1.0%. Caffeic acid was added at 100 ppm to the homogenate. These varying concentrations allowed for an initial screening to assess the efficacy of each antioxidant under different application conditions.

Based on the results of the screening trials, subsequent extractions were performed using the most promising antioxidants, *i.e*. catechins (250 – 2000 ppm) and Duralox® (0.25 – 2.0%), both individually and in combination at ratios of 25:75, 50:50, and 75:25. Antioxidant-free controls were included for comparison.

### 2.3 Protein extraction and quantification

Pea protein was extracted following the protocol described by Karaca et al. (2011). Briefly, pea flour was mixed with water at a 1:10 (*w*.*v*^−1^) ratio. The antioxidants detailed in the previous section were first dispersed in the aqueous phase before suspending the pea flour. The mixture was stirred for 1 h at its native pH, then adjusted to pH 8.5 using 2 M NaOH and stirred for an additional hour. The suspension was centrifuged at 4000×*g* for 20 min at 4 °C, after which the supernatant was separated and acidified to pH 4.5 using 2 M HCl. Following acidification, the mixture was stirred and centrifuged again to collect the precipitated protein. The resulting protein pellet pH was neutralized to pH 7.0, freeze-dried, and stored at −80 °C. All steps were conducted under dim light to minimize oxidative degradation.

### 2.4 Total phenolic compounds

The total phenolic content (TPC) was determined following a standardized procedure described elsewhere by Slinkard and Singleton (1977). Briefly, 0.1 g of each dry pea protein was suspended in 10 mL of methanol:water (70:30 *v*.*v*^−1^) + 1.0% trifluoroacetic acid, sonicated for 5 min, vortexed, and centrifuged (5000×*g*, 5 min, 4 °C). Then, 500 μL of each extract was dissolved in 2.5 mL of Folin–Ciocalteu reagent (10% *v*.*v*^−1^). The mixtures were allowed to stand for 2 min before adding 2 mL of sodium carbonate solution (75 g.L^−1^). After 15 min of incubation at 50 °C, the absorbance of the solutions was measured at 760 nm. For quantification, a calibration curve of gallic acid (10 – 100 μg.L^−1^) was prepared under the same conditions. Results were expressed as μg gallic acid equivalent (GAE) per g of pea protein dry weight (DW).

### 2.5 Quantification of hydroxyoctadecadienoic acids (HODE) and malonaldehyde (MDA) using LC-MS

Lipid oxidation product hydroxyoctadecadienoic acid (HODE), a carboxylic acid-containing oxidation product, was analyzed by developing a method following the principles described by Tullberg et al. (2016) with a modified derivatization procedure, employing 3-nitrophenylhydrazine (3-NPH), a carbodiimide coupling agent, to enhance detection sensitivity for carboxylic acid-containing oxidation products like HODE. First, 3-NPH (MW = 189.6 g mol^−1^) was diluted in LC-MS-grade methanol to a final concentration of 2 mg mL^−1^. This reagent was prepared fresh daily, protected from light, and stored at 4 °C until use to prevent degradation. Similarly, 1-ethyl-3-(3-dimethylaminopropyl)carbodiimide (EDC-6; MW = 191.7 g mol^−1^) was dissolved in LC-MS-grade methanol to a final concentration of 6 mg mL^−1^, with 6% (*v*.*v*^−1^) pyridine, prepared fresh before use, as EDC is moisture-sensitive and can hydrolyze over time. Each pea protein was diluted in water (DF = 5) and vortexed for 10 s to ensure complete homogenization and dissolution of any aggregates. An aliquot of 50 μL of sample was transferred into a 1.5 mL Eppendorf tube, mixed with 50 μL 3-NPH and 50 μL EDC-6 + 6 % pyridine. To facilitate the carbodiimide-mediated coupling reaction between the carboxylic acid group of HODE and 3-NPH, forming a stable nitrophenylhydrazone derivative. The tubes were sealed and shaken for 60 min at 2000 rpm at RT and darkness in a Multi-Tube vortexer (DVX-2500, VWR, USA). After shaking, the tubes were centrifuged (15 000×*g*, 10 min, RT), and 200 μL were collected in a separate 1.5 mL Eppendorf tube. Then, aldehydes were extracted with 500 μL of dichloromethane, the biphasic mixture was vortexed for 30 s and centrifuged (16 000×*g*, 2 min, RT). The extraction step was repeated three times. The supernatants were pooled in a different 1.5 mL Eppendorf tube together and stored at −80 °C until further analysis. For quantification, a standard curve of 13-HODE was prepared in concentrations ranging from 0.005 to 1 μM. The standards were also subjected to derivatization as described above. Targeted LC-MS analysis was performed using a Kinetex C18 column. The mobile phase included 20 mM acetic acid in water (A) and methanol (B). The gradient program was run for 40 min. It started with 70% eluent A and 30% eluent B for 2 min, followed by a linear increase of A to 95% over 8 min. This composition (95% A, 5% B) was maintained for 10 min. From 20 to 25 min, A was further increased linearly to 98% (2% B) and held for 2 min. Eluent A was then reduced linearly to 70% within 1 min, and this ratio (70% A, 30% B) was maintained until the end of the program. Detection used a QTRAP 6500+ mass spectrometer (Sciex, Singapore) in both positive and negative ESI modes. The results were expressed as μg HODE.g^−1^ of pea protein in DW.

Malonaldehyde (MDA), was quantified following the procedure of Tullberg et al. (2016). First, samples were dispersed in Milli-Q water at a 1:5 dilution ratio and homogenized using a vortex for 10 s. Then, 1 mL of each homogenate was transferred into a 1 mL Eppendorf tube and mixed with 20 μL of BHT (0.1 g mL^−1^ in methanol), 40 μL of EDTA (0.02 M in Milli-Q water), and 500 μL of 0.25 M HCl to acidify the mixture. The samples were vortexed for 10 s and allowed to stand for 5 min at RT. The supernatant was separated by centrifugation (16 000×*g*, 2 min, RT), and 400 μL of the supernatant was transferred to a new tube and mixed with 25 μL of 2,4-dinitrophenylhydrazine (DNPH) (2 mg mL^−1^ in methanol). The mixture was vortexed for 10 s and left to stand at RT for 60 min. Aldehydes were then extracted with 500 μL of dichloromethane. The samples were vortexed and centrifuged again (16 000×*g*, 2 min, RT), and the organic phase (bottom layer) was transferred to a new Eppendorf tube. This extraction process was repeated three times in total. After extraction, samples were dried using a SpeedVac and re-dissolved in 250 μL of methanol prior to analysis. For quantification, a standard curve of MDA, prepared in concentrations ranging from 0.3 to 60 μM was prepared and analyzed as described above for HODE. The areas of each sample were compared to those of each MDA concentration.

### 2.6 Spectrophotometric determination of lipid hydroperoxides (LOOH)

Lipid hydroperoxide content was determined following the International Dairy Federation (IDF) method, modified by Shantha and Decker (1994). To extract the lipids, 2 g of each pea protein sample was suspended in 8 mL of a chloroform:methanol mixture (1:2 *v*.*v*^−1^) and vortexed for 5 s. Subsequently, 5 mL of chloroform and 2 mL of 0.5% KCl were added, followed by another 5 s vortex. The mixture was then centrifuged at 10 000×*g* for 10 min. From the resulting chloroform layer, 2 mL of the lipid fraction was collected and combined with 2 mL of a chloroform:methanol solution (7:3 *v*.*v*^−1^). To this, 25 μL each of ammonium thiocyanate and iron (II) solutions were added. After 5 min of reaction time, absorbance was measured at 500 nm. Peroxide values were calculated using a standard curve prepared with iron (III) chloride solution (Soendjaja and Girard, 2024).

### 2.7 Measurement of selected off-flavor related volatile organic compounds via HS-SPME-GC-MS

Selected volatile beany off-flavor associate compounds and the analytical procedure (HS-SPME)-GC–MS were based on the protocol described by Gultekin Subasi et al. (2024). First, 0.5 g of protein isolate was mixed with 5 mL Milli-Q water in 20 mL dark glass vials. Volatile organic compounds were extracted from the headspace using a 75 μm CAR/PDMS SPME fiber at 60 °C for 40 min with stirring (500 rpm), then thermally desorbed and injected splitless into the GC–MS for 5 min.

Analysis was performed on a Shimadzu TQ8030 GC–MS with a ZB-1701 column using helium (1.5 mL min^−1^) as the carrier gas. The GC inlet was set to 300 °C, and the oven temperature ranged from 35 to 260 °C. The MS transfer line and ion source were held at 265 °C and 200 °C, respectively, scanning 30–500 amu. As an internal standard, 8 μL of ethylpyridine (50 mg mL^−1^ in ethanol) was added directly to all samples. Quantification was based on relative peak areas normalized to the internal standard and compared to external calibration standards. The results were expressed as μg of each volatile compound per g of pea protein in DW.

### 2.8 Free radical detection by electron paramagnetic resonance (EPR) spectroscopy

Free radicals were extracted and detected as described by Amft et al. (2020) by modifying and adapting the method to pea protein. Pea protein was suspended in an ethyl acetate:methanol mixture (3:2 *v*.*v*^−1^) at a 1:10 (*w*.*v*^−1^) ratio. This solvent system was selected for its balanced polarity, as ethyl acetate provides moderate non-polar solubility for hydrophobic radical adducts, while methanol enhances extraction of polar components from the protein matrix. To this suspension, 100 μL of 200 mM N-tert-butyl-α-phenylnitrone (PBN) dissolved in ethanol was added. After vortexing for 10 s, the sample was incubated at 50 °C for 30 min in a water bath. This temperature and duration promote radical diffusion and trapping efficiency without causing thermal degradation of the protein or adducts. After incubation, the samples were centrifuged (4000×*g*, 10 min, 4 °C), and the supernatant was separated and evaporated under a nitrogen flow until dryness. Dry samples were resuspended in 150 μL of the same solvent mixture prior to analysis. Microcapillaries (6.5 cm in length, typically borosilicate glass with an inner diameter of ∼1 mm) were filled with 50 μL of the sample, and both ends were sealed with paraffin wax. For measurement, the microcapillary was inserted into an NMR tube. This preparation allows direct insertion into the EPR spectrometer cavity for continuous-wave detection, where hyperfine splitting patterns from the PBN adducts (*e.g.*, characteristic triplets from nitrogen and doublets from beta-hydrogens) can be resolved to quantify specific radical species.

A calibration curve of DPPH dissolved in ethanol (10 μM to 1 mM), each concentration with 100 μL of 200 mM PBN was used to determine spin concentration, with Avogadro's number (6.02214076 × 10^23^ mol^−1^) used in the calculation.

EPR measurements were performed in a Spinscan X-band EPR spectrometer (LINEV Systems, Germany) at 9.40 GHz, 10 mW power, 10 dB attenuation, 200 μT modulation amplitude, and 109 kHz modulation frequency, at RT. Data were processed with e-Spinoza (v1.1.02) and Origin 8.5, using single and double integration of spectra to determine radical line shapes and spin concentration, respectively. The measurements were designed for quantification of radical levels only. Identification of specific radical adducts was not pursued in this study. Results were expressed as the number of spins per gram of isolated protein DW.

### 2.9 Lipoxygenase (LOX) activity

In order to investigate the effect of the antioxidant on LOX activity independent from the effect of the pH-shift induced during the protein extraction, the pea flour was suspended in water at a 1:10 ratio (*w*.*v*^−1^) in the presence of the selected antioxidants mimicking the first step of the protein extraction but the sample was immediately freeze-dried. LOX activity was measured following the protocol described by Gultekin Subasi et al. (2024). First, 0.1 g of sample was mixed with 10 mL phosphate buffer (10 mM, pH 6.5), stirred for 5 h at RT, and the enzyme-rich extract supernatant was collected by centrifugation (9100×*g*, 10 min, RT). The model substrate solution was prepared by mixing linoleic acid (140 μL) and Tween 20 (140 μL) and emulsified into 8 mL of phosphate buffer. Then, the solution was clarified by adding 1.1 mL of 0.5 M NaOH, and the volume was brought to 50 mL with phosphate buffer. The stock substrate solution was diluted (1:40 *v*.*v*^−1^) with 0.1 M sodium borate buffer (pH 9.0). Then, 50 μL of crude enzyme extract was mixed with 1.25 mL of the model substrate. The change in absorbance was recorded at 234 nm for 10 min using a UV spectrophotometer. The unit of LOX activity was U per g of flour, where U is defined as the numeric increase in absorbance per minute, as described in Eq (1).

[Eq. 1] $$\mathrm{LOX}\;\mathrm{activity}\;\left(\frac{\mathrm{unit}}{g}\mathrm{pea}\;\mathrm{flour}.\min\right)=\frac{{\Delta \text{OD}}_{234nm}\times V_{2}}{\Delta t\times V_{1}}\times \frac{V_{0}}{m}$$

where ${\Delta \text{OD}}_{234nm}$ denotes the change in optical density (OD) over the time interval Δt at 234 nm; Δt signifies the cumulative duration of the reaction (min); $V_{0}$ represents the total volume of the crude enzyme extract (mL); $V_{1}$ indicates the specific volume of the crude enzyme extract added (mL); $V_{2}$ denotes the overall volume of the reaction mixture (mL); and m signifies the mass of pea flour (g).

### 2.10 Sensory evaluation

To ensure the integrity of the sensory characteristics, all food-grade samples were freshly prepared immediately prior to analysis to minimize any alterations in flavor that could result from oxidation. The protein samples were dissolved in water at a 5.0% concentration and assessed by a trained analytical sensory panel at Kristianstad University using Quantitative Descriptive Analysis (QDA) (Lawless and Heymann, 2010). The panel consisted of nine assessors during the sensory evaluation's day 1 and due to disease, eight assessors during day 2, selected in accordance with ISO standards 8586:2023 and 6658:2017. Through a structured training process, panelists identified and defined relevant sensory attributes (as outlined in Table 1) by analyzing a range of protein samples that exhibited the most pronounced differences in attributes such as flavor and color, ensuring comprehensive coverage of potential variations. Thereafter panelists received training on applying a continuous line scale, which required reaching a consensus for consistent evaluations. This intensity scale ranged from 0 to 100 mm, with the descriptors “little” and “much” marked at 10 mm and 90 mm, respectively. Sensory evaluations took place in a laboratory compliant with ISO 8589:2010 standards, and data were recorded using EyeQuestion software (version 4.11.68, Logic8, The Netherlands). The QDA was conducted over two sessions, each lasting 2 h on consecutive days, with every sample evaluated in triplicate by each panelist. To mitigate bias, samples were marked with a three-digit code and were presented monadically in a randomized sequence unique to each panelist. Palate cleansing was maintained throughout the sessions using water and neutral wafers between samples.

**Table 1:** Sensory attributes and their definitions.

Category	Attribute	Description
**Odor (O)**	Yellow pea	Cooked
	Pea sprout	
	Sulfur	Compost, cabbage
	Roasted	Towards burnt
**Appearance (A)**	Brown	Color intensity
	White	Color intensity
**Taste (T)**	Saltiness	Basic taste
	Sourness	Basic taste
	Sweetness	Basic taste
	Umami	Basic taste
	Bitterness	Basic taste
**Flavor (F)**	Yellow pea	Raw
	Nuttiness	Roasted
	Pea sprout	Green
	Burnt	Sesame oil
	Yeast/sulfur	
	Black tea	Tannin
	Metal/iron	
**Texture (Tx)**	Viscosity	
	Greasy mouthfeel	
	Rough/dehydrating	

Ethical approval for the present study was obtained from the Ethics Board at Kristianstad University (Ref. U2024-2.3.4-725) prior to its commencement.

### 2.11 Statistical analysis

Experiments were conducted in duplicate, with each sample analyzed at least in triplicate. Principal component analysis (PCA) and heatmap visualization (OriginPro, 2023, v10.0.0.154, 64-bit) were used to compare volatile profiles and their association with sample type and antioxidant concentration. Statistical analyses were performed using OriginPro 2023, v10.0.0.154, 64-bit, including ANOVA at a 95% confidence level. Descriptive sensory data were analyzed using a two-way ANOVA with products and panelists as fixed factors, followed by Tukey's pairwise comparison test (95% confidence level).

## 3 Results and discussion

### 3.1 Screening antioxidants for mitigating lipid oxidation and formation of volatile compounds during protein extraction

Primary oxidation products, such as hydroperoxides (HPODE) and conjugated dienes, provide limited insight into overall oxidative damage due to their short-lived nature. They rapidly degrade and become less reliable indicators at later stages of oxidation (Vangaveti et al., 2010). Therefore, this study primarily focused on tracking formation and accumulation of HODE, which are non-volatile early-stage secondary oxidation products formed via the reduction of HPODE from linoleic acid. These hydroxyl derivatives are more stable and thus provide a more reliable assessment of early lipid oxidation. It should be noted, however, that HODE formation reflects only the oxidation of linoleic acid (18:2). Because linolenic acid (18:3) is even more susceptible to oxidation, relying solely on HODE would overlook key 18:3-derived secondary oxidation products. For this reason, malondialdehyde (MDA), a polar, non-volatile terminal secondary product arising from the degradation of polyunsaturated fatty acids with three or more double bonds (*e.g.*, arachidonic and linolenic acids), analysis was included to ensure broader coverage of lipid oxidation pathways.

**Fig. 2:**
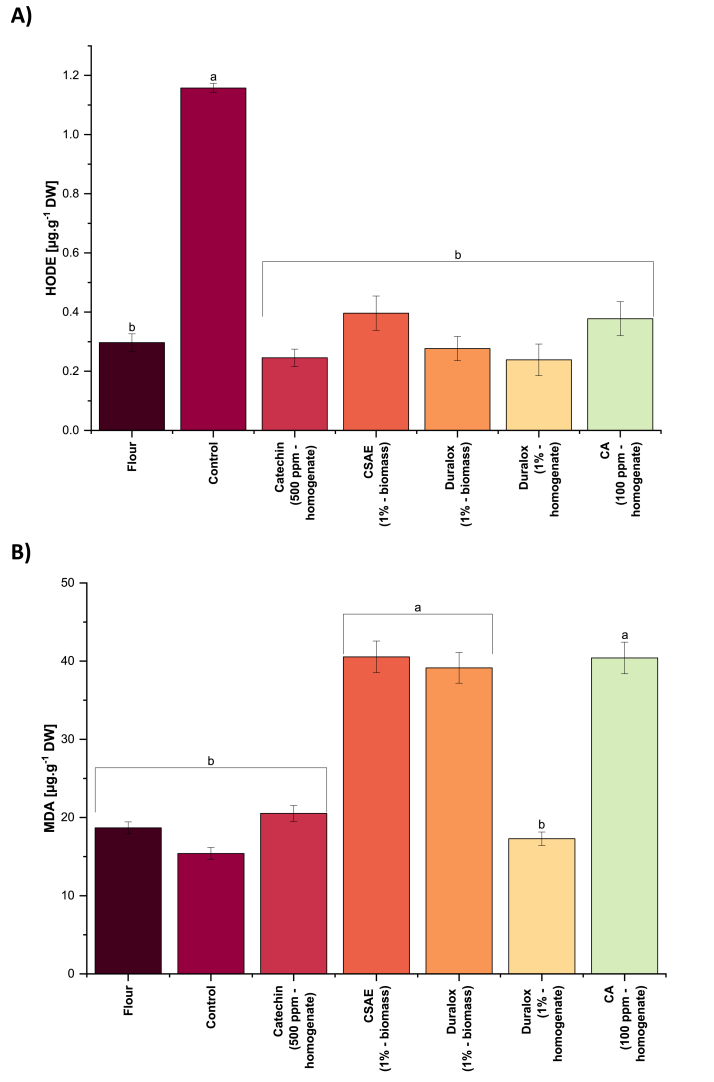
Lipid oxidation products HODE (Fig. 2A) and MDA (Fig. 2B), detected by LC-MS in pea protein. CSAE: coffee spent aqueous extract, CA: caffeic acid. Different small letters represent statistically significant differences across the samples. Different capital letters represent statistically significant differences within the same antioxidant group (Tukey test; *p* < 0.05).

Content of HODE in the protein isolate extracted using the classic alkaline solubilization followed by isoelectric precipitation (*i.e.,* control) (1.15 μg g^−1^ DW) increased 4-fold compared with the initial pea flour (0.3 μg g^−1^ DW) (Fig. 2A). This proves the very high intensity of lipid oxidation during the protein extraction process, which could be the main driver of off-flavor formation in this step, previously reported by Sajib et al. (2023) and Gultekin Subasi et al. (2024). The authors also reported 4-100-fold increase in the quantity of beany volatile compounds, especially hexanal. HODE content in the proteins extracted in the presence of all the added antioxidants was reduced significantly compared to the control, reaching values close to the flour which proves their effectiveness in inhibiting either formation of hydroperoxides or their degradation pathways leading to the formation and accumulation of HODE. Specifically, 500 ppm of catechins and 1.0% Duralox® (based on homogenate, respectively), more effectively reduced HODE formation, with values of ∼0.2 μg g^−1^ DW. In contrast, the control sample, without added antioxidant, exhibited the highest HODE content (1.15 μg g^−1^ DW).

MDA content showed a completely different trend among the samples compared with HODE levels (Fig. 2B). There was no significant difference in MDA content of the flour and protein isolate produced without antioxidant (control) and in the presence of 500 ppm of catechins and 1.0% Duralox® (based on homogenate and biomass, respectively). However, the protein isolate produced in the presence of CSAE, high concentration of (*i.e.*, Duralox 1.0% biomass) and CA showed 2-fold higher MDA compared with the control. The plot also provides insight into the oxidative state of the molecules following antioxidant addition. The seemingly lower MDA content in the control sample, compared to some antioxidant-treated samples, could be attributed to the instability of MDA. Therefore, the lower MDA levels in the untreated sample might result from its ongoing and fast oxidative degradation (Ogugua Victor et al., 2011), while the higher MDA content in the pea protein extracted with some of the added antioxidants may reflect their protective effect in preventing further MDA degradation. Overall, it seems that MDA cannot be a very strong indicator of the lipid oxidation degree during the pea protein extraction compared with HODE and needs to be complemented with other oxidation markers.

The next step focused on evaluating the effect of protein extraction and addition of antioxidants on the formation of volatile organic compounds in the pea proteins to validate their potential in reducing off-lipid oxidation-related beany flavor formation (Supplementary information SS. 1). In total, 13 compounds were targeted with concentrations varying across the different samples. Among them, 3-methyl-1-butanol, 1-pentanol, hexanal, and hexanol were the main compounds in the samples. Four of the quantified volatile compounds, including 1-pentanol, hexanal, 1-octen-3-ol and 1-nonanol, showed 2-6-fold increase in the protein isolated without antioxidant (control) compared with the flour. This is in line with the previous increase in concentration of volatile compounds during pea protein extraction (Gultekin Subasi et al., 2024; Sajib et al., 2023). This again proves the formation or up-concentration of beany volatile compounds during pea protein extraction using the classic protein extraction method.

It is very interesting that the formation or up-concentration of beany volatile compounds followed the magnitude of lipid oxidation detected during the protein extraction by HODE which could again support the hypothesis that lipid oxidation is their origin, as has been recently demonstrated by Sharifi et al. (2026). Notably, 1-octen-3-ol, a key contributor to mushroom-like off-flavors, is formed from the oxidative degradation of linoleic acid through a 10-hydroperoxide intermediate, which can subsequently be cleaved or rearranged by endogenous enzymes such as hydroperoxide lyase or related isomerases (Y. Gao et al., 2025). The modulation of these intermediates by the applied antioxidant treatments likely contributed to the reduced formation of this compound.

The results also reflected the effectiveness of catechins and Duralox® in mitigating the formation of volatile compounds during wet pea fractionation. Notably, catechins at 500 ppm effectively reduced the content of 1-pentanol by 3.5 times, hexanal by 6 times, and hexanol by 1.3 times, compared to the control sample. However, the rest of the antioxidants were not effective in reducing the formation of the volatile compounds.

However, a different trend was observed for 3-methyl-1-butanol, where a significant increase occurred exclusively with catechins addition at 500 ppm (3.8 times). Catechins are known to inhibit lipid peroxidation, a process that leads to the formation of aldehydes such as hexanal and alcohols like hexanol and pentanol (J. Chen et al., 2022). By scavenging free radicals and breaking the chain reactions of lipid peroxidation, catechins reduce the formation of these compounds. Conversely, catechins can interact with other polyphenolic compounds and dietary components, potentially enhancing the formation of certain volatiles like 3-methyl-1-butanol through synergistic effects (J. Yao et al., 2022).

**Fig. 3:**
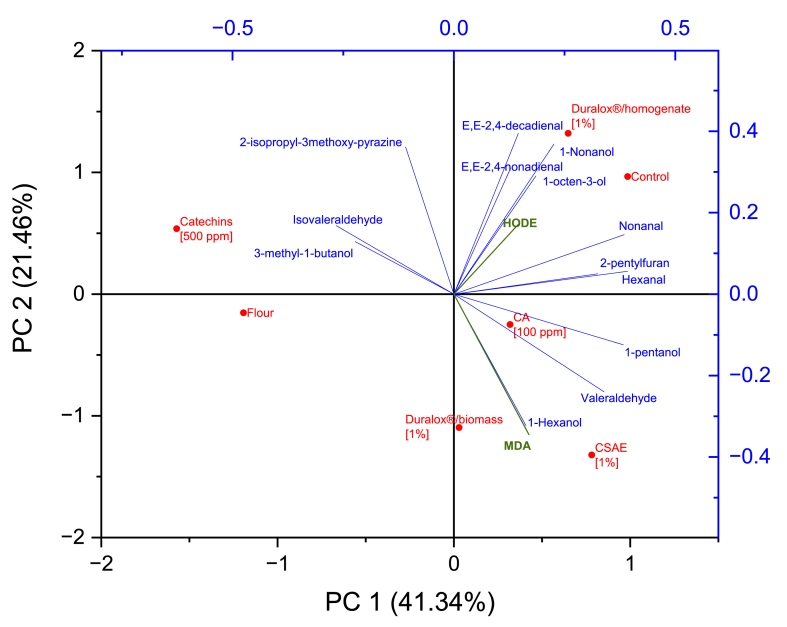
PCA biplot obtained from volatile compounds of pea protein determined by GC–MS. CA: caffeic acid, CSAE: coffee spent aqueous extract.

The variation in volatile organic compounds levels is a sound indication that antioxidants influence the volatiles differently. In this line, PCA was employed to further evaluate the impact of different antioxidants on the volatile compounds’ composition of pea protein. As shown in Fig. 3, PCA enabled clear discrimination among the pea protein samples. In the score plot, the proximity of sample points indicates similarity in their volatile profiles, while the closeness between samples and specific volatiles on the biplot indicates a strong correlation. Principal components PC1 and PC2 accounted for 41.34% and 21.46% of the total variance, respectively, with a cumulative contribution of approximately 63%, providing good discrimination among the treatments.

Along PC1, a clear separation was observed between the control and Duralox® 1.0% homogenate samples (positive side) and the flour and sample enriched with catechins at 500 ppm (negative side). The control sample was located on the positive side of PC1 and PC2, clustered with oxidation markers such as HODE, *E*,*E*-*2*,*4*-decadienal, *E*,*E*-*2*,*4*-nonadienal, and alcohols like 1-nonanol and 1-octen-3-ol, indicating higher levels of lipid oxidation products. In contrast, the flour and catechin-treated sample, located on the negative side of PC1, with a distance from these volatiles, suggesting a protective effect against oxidation when catechins are present.

Regarding the flour, as shown in the analysis of volatiles, MDA, and HODE, it contains significantly lower amounts of these molecules, indicating their concentration increases during protein extraction and further proving that it is mostly driven by lipid oxidation. The flour sample lies in a unique position, suggesting baseline differences in volatile composition relative to treated or control groups. By comparison, caffeic acid and coffee extract exhibited weaker antioxidant activity, as reflected in their proximity to 1-pentanol, valeraldehyde, and 1-hexanol. Meanwhile, the closeness of the flour sample to those enriched with catechins and Duralox® indicates that these antioxidants effectively suppress off-flavor volatiles, bringing levels closer to those naturally present in flour.

Given the distinct effects observed between catechins and Duralox® in the initial volatile compounds profiling, particularly in their ability to reduce specific volatiles, the subsequent analysis aimed to delve deeper into understanding the mechanisms underlying their actions under decreasing concentrations and their mixtures. This next phase of investigation focused on exploring how these antioxidants interact with the pea protein matrix and the volatile compounds. This approach was used to examine whether their behavior is primarily dictated by antioxidant capacity or by the ability to inhibit lipoxygenase activity.

### 3.2 Understanding the effect of antioxidant concentration/combination on off-flavor formation and its mechanism

#### 3.2.1 Effect of antioxidant concentration/combination on the formation of beany volatile compounds

A key factor influencing consumer acceptance of pea protein products is their characteristic beany flavor, which restricts their use in the food industry. The unpleasant odors are caused by volatile compounds originating either inherently from the peas themselves or from the degradation of fatty acids (Gultekin Subasi et al., 2024). In syntony with the observed reduction of oxidation markers in catechins-treated pea protein samples, which are closely associated with increasing TPC, the impact of these antioxidants on selected volatile compounds widely associated with beany flavor in peas via GC-MS was also investigated. As summarized in Table 2, among the total 14 targeted and measured compounds in the samples, with 1-pentanol, hexanal, and 1-hexanol stood out among the various volatiles. Amid these, hexanal had the highest concentration, ranging from 1.2 to 10 μg g^−1^ DW, followed by 1-pentanol (1.4 to 2.6 μg g^−1^ DW) and 1-hexanol (2.6 – 4 μg g^−1^ DW). The concentrations of the remaining compounds did not exceed 3 μg g^−1^ DW.

**Table 2:** Quantitative comparison of volatile compounds (expressed in μg.g^−1^ DW) in pea protein extracted under the presence of catechins and Duralox® performed by HS-SPME-GC-MS.

Volatile compounds [μg.g^−1^ DW]		Isovaleraldehyde	Valeraldehyde	1-Pentanol	Hexanal	1-hexanol	2-pentylfuran	Nonanal
Control		0.03 ± 0.01^f^	0.04 ± 0.03^bcde^	2.09 ± 0.39^abc^	4.23 ± 1.09^bc^	2.72 ± 0.26^cd^	0.07 ± 0.02^c^	0.08 ± 0.02^b^
**Catechins**	**250 ppm**	0.09 ± 0.00^cde^	0.00 ± 0.00^f^	2.38 ± 0.63^ab^	5.95 ± 0.58^b^	2.56 ± 0.22^d^	0.09 ± 0.02^ab^	0.06 ± 0.01^bcd^
	**500 ppm**	0.15 ± 0.05^ab^	0.07 ± 0.07^b^	2.28 ± 1.31^abc^	3.64 ± 3.60^bcd^	3.24 ± 1.18^abcd^	0.08 ± 0.03^abc^	0.03 ± 0.02^defg^
	**750 ppm**	0.10 ± 0.01^cd^	0.06 ± 0.00^bc^	1.63 ± 0.26^bc^	2.04 ± 0.80^cd^	2.87 ± 0.14^bcd^	0.07 ± 0.02^abc^	0.03 ± 0.01^g^
	**1000 ppm**	0.12 ± 0.04^bc^	0.00 ± 0.00^f^	1.68 ± 0.51^bc^	2.06 ± 0.10^cd^	2.63 ± 0.67^cd^	0.07 ± 0.02^c^	0.02 ± 0.00^g^
	**2000 ppm**	0.17 ± 0.05^a^	0.16 ± 0.00^a^	1.39 ± 0.51^c^	1.23 ± 0.41^d^	3.29 ± 0.60^abcd^	0.06 ± 0.02^c^	0.03 ± 0.00^g^
**Duralox®**	**0.25%**	0.05 ± 0.00^ef^	0.00 ± 0.00^f^	2.63 ± 0.52^a^	5.36 ± 0.87^b^	3.05 ± 0.38^bcd^	0.10 ± 0.02^a^	0.11 ± 0.03^a^
	**0.50%**	0.04 ± 0.00^f^	0.03 ± 0.00^cdef^	1.81 ± 0.16^abc^	6.14 ± 0.39^b^	3.08 ± 0.09^bcd^	0.07 ± 0.00^bc^	0.05 ± 0.00^cdef^
	**1.00%**	0.04 ± 0.01^f^	0.01 ± 0.01^ef^	2.52 ± 0.83^ab^	8.84 ± 2.73^a^	3.51 ± 0.64^abc^	0.08 ± 0.02^abc^	0.06 ± 0.02^bc^
	**1.50%**	0.04 ± 0.01^f^	0.02 ± 0.02^def^	2.23 ± 0.42^abc^	8.84 ± 2.26^a^	3.97 ± 0.83^a^	0.08 ± 0.01^abc^	0.06 ± 0.01^bcde^
	**2.00%**	0.05 ± 0.00^f^	0.00 ± 0.00^f^	1.86 ± 0.05^abc^	10.48 ± 0.16^a^	3.66 ± 0.07^ab^	0.08 ± 0.00^abc^	0.06 ± 0.00^bc^
**25% catechins + 75% Duralox®**		0.06 ± 0.01^ef^	0.06 ± 0.01^bcd^	1.62 ± 0.18^bc^	3.84 ± 1.70^bcd^	2.76 ± 0.33^cd^	0.06 ± 0.01^c^	0.03 ± 0.01^efg^
**50 % catechins + 50% Duralox®**		0.06 ± 0.01^def^	0.03 ± 0.00^def^	2.05 ± 0.28^abc^	4.13 ± 0.98^bc^	3.32 ± 0.49^abcd^	0.06 ± 0.00^c^	0.03 ± 0.00^g^
**75 % catechins + 25% Duralox®**		0.06 ± 0.01^def^	0.05 ± 0.01^bcd^	1.63 ± 0.17^bc^	5.14 ± 1.53^b^	3.11 ± 0.18^abcd^	0.06 ± 0.00^c^	0.03 ± 0.01^fg^

Data is represented as average ± SD. Values with different letters in a column represent significant differences (p < 0.05) (*n* = 2).

When analyzing the concentration of individual elements as a function of the antioxidant used during extraction, it is evident that catechins significantly mitigated the production of off-flavor-associated volatile compounds. Moreover, this mitigation was directly proportional to the concentration applied. When catechins were included in the extraction medium, the concentration of hexanal decreased from 6 to 1.2 μg g^−1^ DW, with catechins concentrations ranging from 250 to 2000 ppm, respectively. In contrast, when Duralox® was used, the hexanal concentration ranged from 5.4 to 10.5 μg g^−1^ DW, demonstrating its ineffectiveness compared to catechins alone. In all cases, a decrease in volatile organic compounds’ concentration was observed with increasing antioxidant concentrations.

**Fig. 4:**
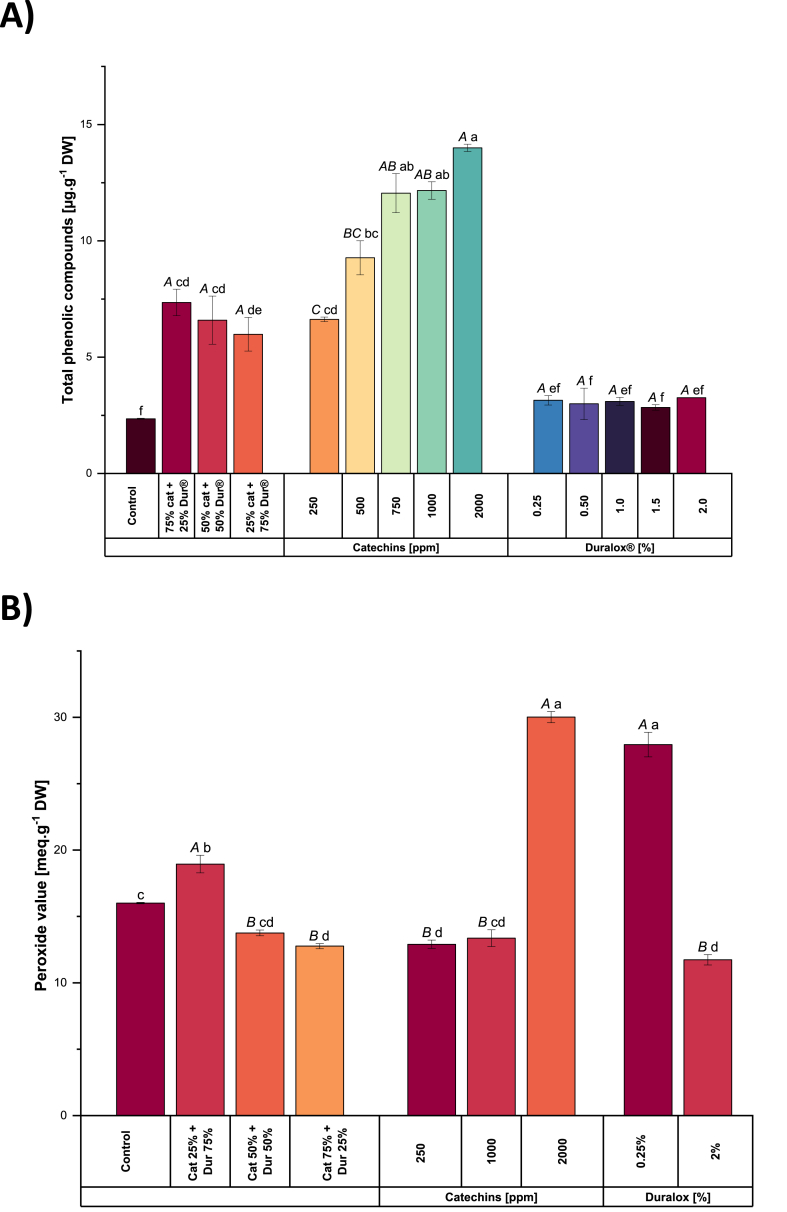

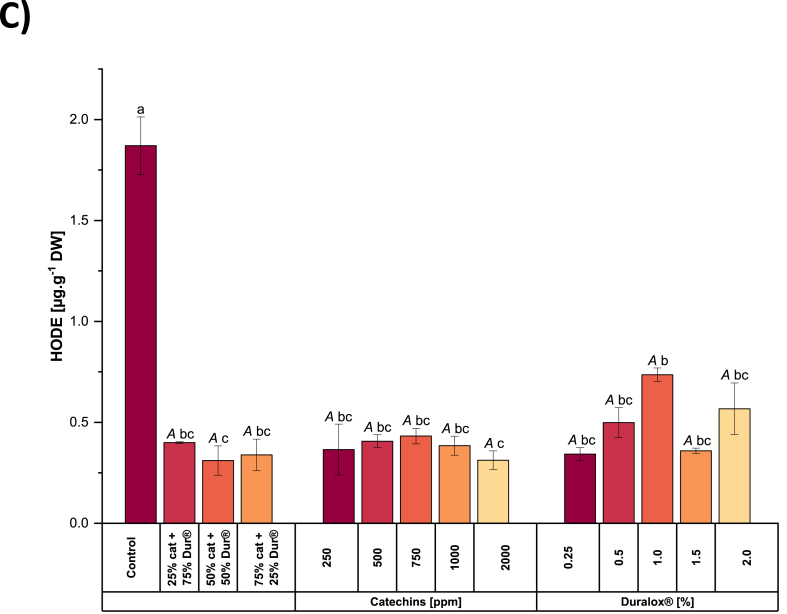
Total phenolic content (TPC) (4A), Peroxide value (4B) and hydroxyoctadecadienoic acids (HODE) content (4C) in pea protein extracted with catechins, Duralox® or their combinations. Different small letters represent statistically significant differences across the samples. Different capital letters represent statistically significant differences within the same antioxidant group (Tukey test; *p* < 0.05). Cat = catechins, Dur = Duralox®.

Notably, this trend closely mirrored the increase in TPC, which rose from 6.6 to 14 μg g^−1^ DW with increasing catechins levels, while remaining around 3 μg g^−1^ DW in all Duralox®-treated samples (see Fig. 4A). In the samples treated with mixed catechins + Duralox®, the TPC decreased from 7.4 to 6 μg g^−1^ DW with decreasing catechins content. This correlation proves that TPC plays a crucial role in modulating volatile profiles, as higher TPC was associated with reduced formation of aldehydes and alcohols. Research on the effects of polyphenols on the formation of off-flavor compounds supports our findings. Several studies indicate that polyphenols exhibit dual modes of action. On one side, these molecules inhibit LOX activity by reducing the ferric form of the enzyme to its inactive ferrous form and by chelating the iron at the active site (Ha et al., 2010). On the other side, Yi et al. (2020) observed a marked decrease in lipid oxidation in whey-protein-stabilized emulsions prepared with anthocyanins, polymeric molecules with similar structure and antioxidant properties to those of catechins, largely through their free radical scavenging ability.

During plant protein extraction, cell disruption releases fatty acids from membrane lipids along with oxidative enzymes such as LOX and lipase, while simultaneously exposing them to oxygen, shear forces, heat, and pH shifts that accelerate lipid oxidation (Gultekin Subasi et al., 2024). LOX catalyzes the conversion of linoleic and linolenic acids into lipid hydroperoxides, which hydroperoxide lyase then cleaves into C6 aldehydes, alcohols, and esters known as green leaf volatiles, while lipase activity increases oxidation susceptibility by liberating free fatty acids. In parallel, non-enzymatic autooxidation proceeds via radical chain reactions, generating aldehydes, ketones, and furans that contribute to beany, grassy, or rancid off-flavors (Lampi et al., 2020). Protein isolation from the plant matrix often concentrates these volatiles in the final product relative to the initial biomass, mainly due to processing steps such as alkaline extraction, centrifugation, and removal of fiber or starch.

In this context, the chemical structure of catechins enables multiple protective actions. Catechins inhibit LOX noncompetitively by binding strongly to sites away from its active site, altering enzyme conformation and reducing activity. They interact near tyrosine and tryptophan residues through hydrogen bonding and other stable forces, forming a stable catechins–LOX complex. Their antioxidant properties further suppress LOX-mediated oxidation by scavenging free radicals, and at low concentrations, they act synergistically with other polyphenols such as quercetin for stronger inhibition (Zhao et al., 2020). Catechins have proven effective in radical scavenging in food systems, in line with their polyphenolic structure (Soendjaja and Girard, 2024). In food systems, catechins also effectively trap dicarbonyl compounds, which are key intermediates in lipid oxidation. The pyrogallol motif on the β-ring is critical for both their pro-oxidant potential and dicarbonyl trapping, with catechins containing this motif showing greater efficacy in forming protein carbonyls and trapping dicarbonyls. Moreover, catechins with a galloyl group exhibit enhanced trapping capacity compared to those without, as this feature increases both binding affinity and stability of catechin–dicarbonyl adducts (Ishii et al., 2010; Niemeyer and Brodbelt, 2007).

On the other hand, isovaleraldehyde and 3-methyl-1-butanol showed a significant increase alongside the rise in catechins content. In the first case, isovaleraldehyde increased from 0.13 to 1.37 μg g^−1^ DW, while 3-methyl-1-butanol increased from 7.0 to 16.13 μg g^−1^ DW. This increase can be explained by catechins-induced modifications of protein structure. Catechins bearing catechol or pyrogallol rings can be oxidized under physiological or processing conditions to electrophilic quinones, which readily react with nucleophilic sulfhydryl groups on cysteine residues (R. Chen et al., 2011). Such covalent modification and cross-linking can disrupt secondary and tertiary structures, shifting the balance between α-helix, β-sheet, and random coil, and altering protein flexibility and surface exposure of residues (Bekhit et al., 2026). These conformational changes can modify or disrupt binding pockets that normally retain volatile aroma compounds, thereby altering their partitioning and facilitating their release into the headspace, including compounds such as isovaleraldehyde and 3-methyl-1-butanol (Ishii et al., 2010). In addition, catechins oxidation and thiol modification can influence redox balance and promote secondary oxidation reactions, generating reactive intermediates that contribute to the formation of volatile aldehydes and alcohols. Furthermore, isovaleraldehyde can be reduced to 3-methyl-1-butanol, providing a complementary pathway for the observed increase in the latter compound (Matheis et al., 2016).

Overall, catechins and Duralox® demonstrated to be effective in reducing the content of volatile compounds in pea protein. The reduction rate is concomitant with the individual concentration as well as the mixing ratio in their combinations.

#### 3.2.2 Effect of antioxidant concentration/combination on the formation of hydroperoxides and HODE

Lipid hydroperoxides are organic compounds containing a hydroperoxyl group (ROOH) that arise from lipid oxidation. In plants, they are primarily generated through the LOX pathway, in which polyunsaturated fatty acids are oxidized to form lipid hydroperoxides. These hydroperoxides serve as precursors to a range of volatile compounds, including aldehydes, ketones, and alcohols, that contribute to the characteristic aroma and flavor of fruits and vegetables (Tang et al., 2024).

The peroxide value (PV) measured in pea protein extracted without antioxidants was ∼16 mEq.g^−1^ DW (Fig. 4B). This moderate value likely reflects a steady-state balance between the formation and degradation of lipid hydroperoxides (ROOH), with no substantial accumulation. In this scenario, the PV measurement may have been taken when ROOH breakdown was actively occurring rather than during their peak formation. This interpretation aligns with the high LOX activity observed (see Fig. 6), which strongly drives the enzymatic initiation of lipid oxidation. In addition, non-enzymatic radical pathways contribute to ROOH formation. The hydroperoxides generated are rapidly decomposed into secondary oxidation products, including HODE and volatile compounds such as hexanal. As a result, both HODE levels and off-flavor volatiles are elevated, yet PV remains in a mid-range because ROOH are continuously converted into downstream oxidation products.

In the catechins-treated sample at 2000 ppm, the PV is higher than in the control (∼30 mEq.g^−1^ DW). This seemingly high value reflects the activity of catechins to scavenge peroxyl radicals (Sroka and Cisowski, 2003), preventing ROOH from breaking down. This stabilizing effect has also been evidenced in other studies that have demonstrated that catechins can slow down the rate of LOOH formation and extend the lag phase before significant lipid peroxidation occurs. This is evident in the reduced formation of conjugated diene hydroperoxides and carbonyl compounds in the presence of catechins (Lam et al., 2005). Besides that, catechins modulate the activity of pro-oxidant enzymes, as will be described in the coming sections.

However, at concentrations below 2000 ppm, catechins are less effective at preventing oxidation, as indicated by the peroxide value (12.9 mEq.g^−1^ DW). In this case, the PV reflects a reaction process that is still progressing, although at a slower rate than in the control. Additionally, catechins can exhibit pro-oxidant activity under certain conditions, promoting the formation of protein carbonyls through Strecker-type reactions. This pro-oxidant potential is influenced by catechins structure, with pyrogallol-type catechins and those containing a galloyl group showing higher activity (Ishii et al., 2010).

With Duralox® at 0.25%, the PV is also higher than in the control (27.9 mEq·g^−1^ DW), but very likely due to different mechanistic reasons. Duralox®, a rosemary/tocopherol-based antioxidant, partially inhibits LOX (see Fig. 6) and slows the breakdown of ROOH, leading to their accumulation and a higher PV. However, its phenolic components can reduce Fe^3+^ to Fe^2+^, enhancing metal-driven redox cycling (Reis et al., 2006). Thus, while Duralox® appears to reduce some markers of lipid oxidation, its interaction with transition metals under wet fractionation conditions introduces a pro-oxidant element, leading to a less comprehensive suppression of oxidation compared to catechins.

Regarding the combination of catechins and Duralox®, when Duralox® was present at a higher proportion (75%), the peroxide value (PV) reached its highest levels (18.9 mEq.g^−1^ DW), likely due to the combined effect of partial inhibition of lipid oxidation and the accumulation of hydroperoxides (ROOH) that were not readily decomposed. Duralox® slowed ROOH breakdown but, through its phenolic components, can also promote metal redox cycling in the aqueous protein matrix, sustaining ROOH formation and increasing free radicals, as already explained. At the same time, the low catechins content in this ratio meant weaker LOX inhibition and less suppression of both enzymatic and non-enzymatic initiation pathways, leading to continued hydroperoxide production. In contrast, when catechins dominated (75%), PV decreased (12.7 mEq·g^−1^ DW), which could be due to catechins strongly inhibited LOX, effectively quenched radicals, and reduced ROOH formation at the outset. This dual-mode antioxidant activity limited both enzymatic and free-radical oxidation, resulting in lower HODE, while the reduced proportion of Duralox® minimized any potential metal-mediated pro-oxidant effects.

**Fig. 5:**
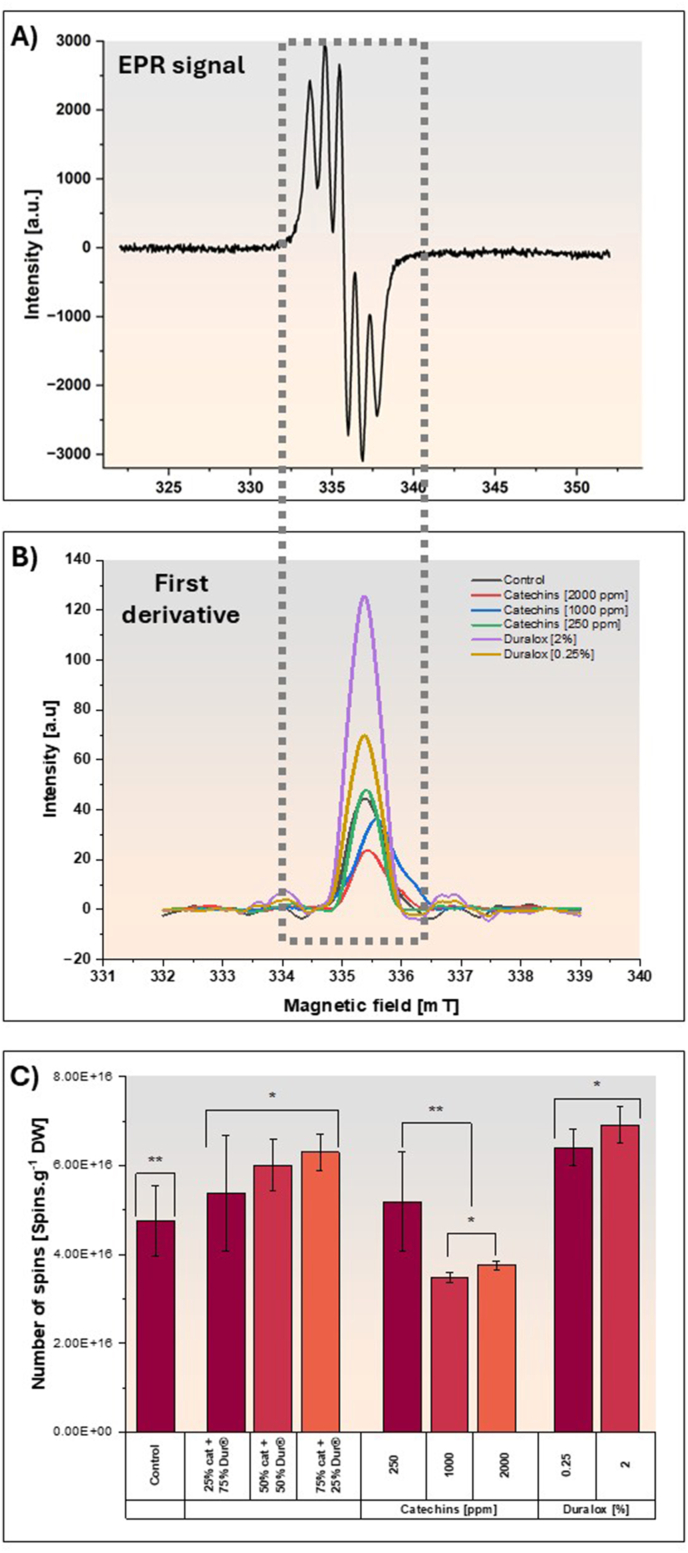
EPR example signal (A), the first derivative of the signal and represents the relative abundance of free radicals, calculated using the second derivative and Avogadro's number (C) for the pea protein isolates extracted in the presence of different antioxidants and concentrations. Cat means catechin and Dur means Duralox®.

Fig. 5C illustrates the effect of antioxidant type and concentration on HODE levels. In control samples, HODE concentrations reached around 1.8 μg g^−1^ DW, indicating robust early-stage lipid peroxidation. This high content suggests significant free radical-mediated activity in the absence of antioxidant intervention, consistent with literature identifying HODE as a sensitive indicator of initial oxidative stress in biological and food systems (H. Zhang et al., 2025). In samples extracted under the presence of catechins, HODE levels dropped markedly to approximately 0.3 μg g^−1^ DW. This biochemical shift parallels the increases in TPC observed under catechins treatment, where phenolic content rose from 6.6 to 14 μg g^−1^ DW. The elevated antioxidant capacity indicated by higher TPC likely contributed to the suppression of early peroxidation events and modulated the oxidative pathway, reducing primary oxidation products (Puchau et al., 2010). This shift suggests that catechins, known for their radical-scavenging and metal-chelating properties, effectively suppress early-stage peroxidation, reducing HODE formation (Chung et al., 2004; Sroka and Cisowski, 2003). Increasing the concentration of catechins did not result in a significant change in HODE quantity. The presence of catechins seems to mitigate the oxidation process through their unique polymeric phenolic structure, particularly the hydroxyl groups at positions C5 and C7 on the A-ring and C3′ and C4′ on the B-ring (Jin and Yoshioka, 2005). These structural features enable efficient electron delocalization and stabilization of reactive intermediates. Additionally, as stated above, catechins also effectively diminish the catalytic activity of LOX.

**Fig. 6:**
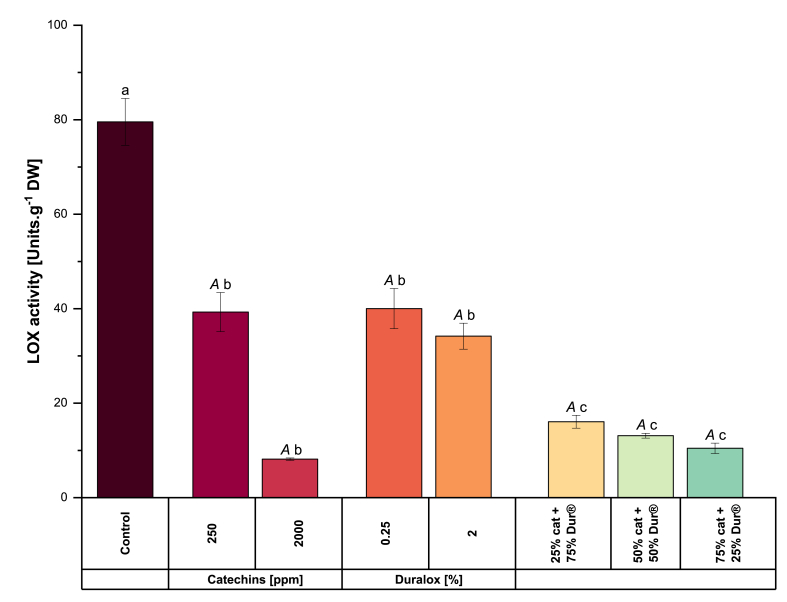
Lipoxygenase (LOX) activity in pea protein extracted with catechins, Duralox® or their combinations. Different small letters represent statistically significant differences across the samples. Cat means catechin and Dur means Duralox®. Different capital letters represent statistically significant differences within the same antioxidant group (Tukey test; *p* < 0.05).

Samples treated with Duralox® exhibited HODE levels around 0.42 μg g^−1^ DW. These moderate and balanced values occurred in samples where TPC remained low (∼3 μg g^−1^ DW), supporting the idea that phenolic content is a critical determinant of antioxidant effectiveness during lipid peroxidation. Beyond its phenolic contribution, Duralox®, a blend of rosemary extract, ascorbic acid, tocopherols, and citric acid, likely suppresses HODE formation through direct inhibition of LOX. In plants, LOX inhibition is commonly mediated by compounds such as polyphenols, flavonoids, and other antioxidants that bind to the enzyme's active site or alter its catalytic conformation, thereby blocking substrate access. Rosemary polyphenols like carnosol may act via this mechanism, providing a complementary pathway to radical scavenging in limiting lipid oxidation (Redrejo-Rodriguez et al., 2004).

Direct studies on lipid peroxidation specifically during wet fractionation of pulses are limited. However, research has shown that endogenous lipids can accumulate in protein fractions during processing, which may increase their susceptibility to oxidation if not properly managed (Koomen et al., 2025). To our knowledge, the study by Gläser et al. (2021) is the only one focusing on HODE generation in pea protein isolates, identifying it as a contributor to bitter taste. They quantified four HODE isomers, in pea protein isolates, with concentrations ranging from 1.2 to 130.44 μg g^−1^ DW. Notably, the control sample in our study is close to the lowest value previously reported. Additionally, similar protective effects have been observed in pea and soy protein solutions with the addition of catechins, tannic acid, grape seed extract, and green tea extract (Soendjaja and Girard, 2024).

Although the structure and bioactivity of catechins are well established, various mechanisms have been proposed to explain their antioxidant activity. As phenolic compounds, catechins satisfactorily mitigate oxidative damage by targeting multiple pathways involved in lipid oxidation and protein interactions. One key mechanism involves the trapping of reactive carbonyl species like glyoxal, which are intermediates in the formation of advanced glycation end-products, including HODE. By sequestering glyoxal, catechins prevent it from reacting with lipids to form undesired oxidation products (Han et al., 2019). Additionally, catechins can undergo self-oxidation to form quinones, which subsequently react with other biomolecules to alleviate oxidative stress. For instance, the formation of catechin-quinones has been documented in plants exposed to ozone layers, where they contribute to redox balance and oxidative stress tolerance (Pheomphun et al., 2019). Besides this, catechins may also suppress oxidation by chelating pro-oxidant metal ions, thereby limiting Fenton-type reactions that accelerate lipid degradation (Pospíšil, 2016). When combined with other polyphenolic compounds, catechins can exhibit synergistic effects, leading to enhanced inhibition of lipid oxidation and reduced HODE formation. Their interactions with proteins further influence antioxidant performance. This interaction helps stabilize proteins and prevents the formation of lipid oxidation products that contribute to off-flavors (Soendjaja and Girard, 2024).

In general, the molecular configuration of catechins not only amplifies their antioxidant potential but also facilitates the formation of stable adducts, effectively disrupting lipid peroxidation and reducing HODE formation.

#### 3.2.3 Effect of antioxidants concentration/combination on free radicals

EPR enables the quantification of free radicals and, under specific conditions, can also assist in identifying radical species (Xu et al., 2020). However, accurate identification requires complementary techniques and advanced spectral analysis. Therefore, this study focused solely on quantification to avoid potential misinterpretations.

Fig. 5A displays the EPR signal, its first derivative (5B) giving a tentative indication of the total population of free radicals, and the calculated relative radical abundance (Fig. 5C). Although the spectra were recorded over a broad magnetic field range (322–355 mT), the quantification of radical abundance was based on the integral of the first derivative signal. This calculation was performed individually for each sample using the value corresponding to the flat region at the end of the curve, where the signal stabilizes. The EPR-measured free radical abundance, ranging from 3.47 to 6.91 × 10^16^ spins·g^−1^ DW, quantifies the radical abundance in pea protein.

EPR measurements revealed a dose-dependent decrease in free radical abundance with catechins addition, with the highest catechins concentration of 2000 ppm resulting in the lowest detected spin numbers (∼3.64 × 10^16^ spins.g^−1^ DW) compared with 250 ppm catechins reported a similar value (∼4.97 × 10^16^ spins.g^−1^ DW) as the control. This reduction in free radical presence aligns with significantly lower concentrations of HODE (∼0.3 μg g^−1^ DW) and key aldehydic volatiles, particularly hexanal (∼1.2 μg g^−1^ DW), in catechins-treated samples. The consistent reducing effect and trend observed across free radical quantification, HODE levels, and volatile compound profiles for the proteins extracted in the presence of catechins confirm that its effective radical scavenging activity significantly contributes to its outstanding mitigative effect on lipid oxidation during the wet fractionation.

During the oxidative process, lipids such as linoleic acid undergo oxidation due to exposure to oxygen, heat, metals, and enzymes like lipoxygenases. This process generates alkyl radicals (R•), alkyl peroxyl radicals (ROO•), and alkoxyl radicals (RO•) (Y. Yao et al., 2024) which lead to the formation of hydroperoxides (LOOH). These hydroperoxides subsequently decompose into various secondary products, including HODE and volatile compounds. Treatment with catechins led to a marked reduction in all oxidative indicators, including HODE, hexanal, and EPR-detected free radicals during pea protein extraction. This shows that catechins effectively suppressed both the initiation and propagation phases of lipid oxidation. As flavanols with strong radical-scavenging activity, catechins likely quenched lipid alkyl and peroxyl radicals by donating hydrogen atoms, thereby reducing the accumulation of free radicals and limiting the formation of lipid hydroperoxides (Zhao et al., 2020). The lower HODE content indicates effective inhibition of primary lipid oxidation, while the reduced hexanal levels confirm that secondary lipid degradation was also curtailed. These results are consistent with catechins' known behavior as a potent chain-breaking antioxidant, particularly effective in hydrophilic environments such as aqueous protein extraction systems (Ishii et al., 2010). It is worth noting the role of lipoxygenases, which catalyze linoleic acid oxidation to HODE which were not fully inhibited by catechins (see next section) which may explain not achieving a complete inhibition of the lipid oxidation chain.

In contrast, additional and increasing Duralox® concentrations seem to induce higher accumulation of free radicals, as observed in the higher spin content for the sample with 2.0% concentration (6.91 × 10^16^ spins.g^−1^ DW), which is 1.1 times higher than the sample with the lowest Duralox® content. Duralox® treatments also exhibited effective but intermediate HODE (∼0.42 μg g^−1^ DW), and elevated volatile organic compounds (5.4 – 10.5 μg g^−1^ DW) levels, reflecting weaker antioxidant activity, consistent with the lower TPC (∼3 μg g^−1^ DW). This trend aligns with the results observed in the PCA biplot (Fig. 3), where Duralox®-treated samples clustered near lipid-derived aldehydes, suggesting a limited ability to inhibit off-flavor formation.

This apparent contradictory behavior of Duralox® in reducing HODE formation by promoting radical accumulation and formation of volatile compounds suggests that it may be related to its effect on LOX activity or it has altered the oxidation pathway rather than fully inhibiting it. The reduction in HODE could be attributed to a reduction of LOX activity by 50% as seen in Fig. 6 or accelerated decomposition of lipid hydroperoxides or diversion of oxidation toward secondary breakdown products.

The elevated EPR spin number likely reflects not only increased radical formation but also the accumulation of relatively stable antioxidant-derived radicals, particularly from rosemary phenolics or tocopherols present in the blend. These antioxidants can form semi-stable radical intermediates, and thus the detected radical population may include both lipid-derived propagating radicals and antioxidant-derived non-propagating radicals. This distinction is important for correctly interpreting Duralox®’s mode of action. However, while antioxidant-derived radicals may contribute to the EPR signal, such species do not propagate lipid oxidation and would typically decrease, rather than increase, volatile breakdown products. The observed rise in volatile compounds, therefore indicates ongoing lipid radical chain reactions, supporting the interpretation that oxidation is not fully suppressed by Duralox® and may instead be redirected toward secondary decomposition pathways.

It is worth noting that Duralox® has shown efficacy in controlling lipid oxidation in fish products, likely due to the different oxidation mechanisms involved. Fish lipids are especially susceptible to hemoglobin-mediated oxidation, which Duralox® effectively inhibits (Wu et al., 2021). In contrast, plant-based systems generally lack hemoglobin, (apart from specialized forms such as leghemoglobin), and since lipid oxidation in these matrices proceeds mainly through non-heme pathways, the antioxidants in Duralox® may be less effective. Furthermore, ascorbic acid, present in Duralox® (Wu et al., 2021), may exhibit pro-oxidant behavior in the presence of transition metals reducing Fe^3+^ to Fe^2+^, thereby catalyzing Fenton-type reactions and increasing radical formation (Reis et al., 2006), which may explain its reduced efficacy or even adverse effects in pea protein.

Mixed catechins-Duralox® treatments reflect Duralox® dominance over radical formation and neutralization behavior. In controls, unmitigated radicals (4.75 × 10^16^ spins·g^−1^ DW) drive high peroxidation, explaining elevated HODE and volatile organic compounds. The seemingly lower free radical content in the control sample, compared to some antioxidant-treated samples, could be attributed to the instability of free radicals. Therefore, the higher spin numbers content in the pea protein extracted with antioxidants may reflect their protective effect in preventing further degradation. In contrast, the lower spin number levels in the untreated sample might result from ongoing oxidative degradation.

Despite its relevance, to our knowledge, no previous studies have applied EPR to evaluate radical activity during antioxidant-assisted pea protein extraction. Therefore, the results herewith delivered offer novel contributions by integrating EPR with chemical markers like HODE and volatiles, allowing for a more comprehensive understanding of antioxidant mechanisms and oxidative dynamics in plant protein systems. Considering these free radical dynamics, the addition of PBN into the system significantly enhanced free radical detection. PBN reacts with short-lived free radicals (*e.g.*, ROO•, RO•) to form stable nitroxide adducts, which have longer half-lives and produce distinct EPR signals, improving sensitivity and allowing for the identification of specific radical species present during yellow pea protein extraction (Amft et al., 2020). This is particularly useful considering that free oxygen-centered radicals produced during lipid oxidation are short-lived, which makes it impossible to detect them directly at RT (Y. Yao et al., 2024). This enhanced detection could reveal the extent of lipoxygenase activity or metal-catalyzed oxidation, offering insights into why catechins reduce radicals, and how Duralox® influences the process, ultimately aiding in optimizing antioxidant strategies to minimize HODE and volatile off-flavors.

On the other hand, the relatively large standard deviations observed in the EPR measurements can be attributed to several factors inherent to both the analytical technique and the sample matrix. First, pea protein extracts are complex, heterogeneous systems that can vary in composition, moisture content, and antioxidant distribution, all of which influence radical generation and stabilization. Second, EPR is highly sensitive to subtle changes in sample handling, packing density within the tube, and temperature fluctuations during measurement, which can lead to signal variability even under controlled conditions. Additionally, the biological variability associated with radical formation and antioxidant interactions further contributes to this spread in values (Mazur, 2006). Despite these challenges, the trends observed across replicates were consistent, and differences between treatments were sufficiently robust to allow reliable interpretation of antioxidant effects.

#### 3.2.4 Effect of antioxidants concentration/combination on LOX activity

Lipoxygenase (LOX) is an enzyme responsible for catalyzing the oxidation of polyunsaturated fatty acids, particularly linoleic acid, which leads to the development of undesirable off-flavors in legumes such as peas (Y. Yao et al., 2024). This enzymatic activity plays a key role in postharvest physiology, as the oxidation process produces secondary metabolites that can negatively impact the sensory quality and consumer acceptance of alternative protein products (Trigo et al., 2024). The extent of LOX activity and the resulting off-flavors vary among different pea cultivars, with genotype having a greater influence than biotic stress (Gultekin Subasi et al., 2024). According to the same authors, the inherent LOX present in the storage parenchyma cells of pea cotyledons becomes activated immediately after the peas are ground into flour, with peak LOX activity occurring when the flour is dispersed in an aqueous medium, particularly at neutral or alkaline pH levels. In this context, wet protein extraction methods such as alkaline solubilization and isoelectric precipitation can significantly affect LOX activity in pea proteins, as these methods often lead to protein denaturation and irreversible aggregation, which can alter the enzyme's function (Zhou et al., 2025). Considering these aspects, this study evaluated LOX activity in flour suspended in water with antioxidants, with no pH shift applied.

Regarding the LOX concentration in our samples (Fig. 6), the study of Gultekin Subasi et al. (2024) delivers values of around 100 units LOX.g^−1^ of pea flour, which is slightly higher than the values reported for samples treated with antioxidants in this manuscript, but close to the control sample. This study's outcomes align with (Bi et al., 2022), who demonstrated that including epigallocatechin-3-gallate significantly mitigates the oxidative activity of LOX.

When catechins and Duralox® were included individually, both reduced LOX activity to half (∼38 LOX units.g^−1^ DW) compared with the control (79.56 LOX units.g^−1^ DW) but no significant differences were observed between 250 ppm catechins and Duralox® inclusion at 0.25% and 2.0%. At the extremes, 2000 ppm catechins mitigated the enzymatic activity to the lowest, with an observed value of 8.16 LOX units.g^−1^ DW resulting in a 10-fold reduction. However, increasing Duralox® concentration to 2.0% did not decrease LOX activity compared with its 0.25% addition. The catechins–Duralox® mixtures exhibited a decreasing trend in LOX content with increasing catechins concentrations (16.08 – 10.45 LOX units.g^−1^ DW), with the lowest value recorded at 75% catechins content.

The correlation between catechins presence and LOX inhibition in the pea protein can be attributed to several mechanisms previously reported. The external application of antioxidants has been shown to suppress enzyme activities induced by oxidative stress, including that of LOX. Cao et al. (2020) demonstrated that phenolic compounds selectively bind near the LOX active site through hydrogen bonding, hydrophobic interactions, and van der Waals forces, altering its secondary structure, and consequently its activity. Other researchers have demonstrated that phenolic compounds can non-competitively inhibit LOX by reducing the active state iron in the enzyme to its ferrous state, thus preventing the activation of the resting enzyme (Mahesha et al., 2007). In the same study, the authors also proposed that antioxidants inhibit LOX activity by scavenging free radicals, which are essential for LOX-mediated oxidation. This action prevents the formation of lipid hydroperoxides, which are products of LOX activity. These effects, regardless of the concentration, can manifest in complete enzymatic activity mitigation or a reduction in the maximum reaction velocity (V_max_) (Ito et al., 2013).

This LOX activity profile is closely linked to EPR, volatile, and HODE data, reflecting the oxidative dynamics during yellow pea protein extraction. The control's high LOX activity corresponds to elevated free radical levels (inferred from typical untreated samples) and increased HODE formation, as LOX catalyzes linoleic acid oxidation to hydroperoxyoctadecadienoic acids (HPODE), which are reduced to HODE or degrade which led to subsequent volatile production (*e.g.*, hexanal, 1-hexanol) as seen in the PCA biplot (Fig. 3). Importantly, LOX activity was reduced by approximately tenfold with 2000 ppm catechins, indicating that inhibition of the enzymatic oxidation pathway plays a central role in catechins' effectiveness. Since HODE is primarily generated via LOX-catalyzed reactions, its significant reduction aligns with the enzymatic inhibition. However, the concurrent decline in EPR spin count, representing free radical levels, suggests that catechins also contributes to non-enzymatic control via radical scavenging or metal ion chelation. The near-total suppression of both primary and secondary oxidation products, along with reduced radical presence, indicates that while LOX inhibition appears to be the dominant mechanism, catechins also interfere with radical propagation and metal-catalyzed pathways, which collectively amplify its protective effect. Therefore, catechins' multi-targeted action, strongly inhibits LOX while attenuating non-enzymatic radical-mediated oxidation, positions it as a promising strategy for oxidative stabilization in legume-based protein extraction systems.

Mixtures like 75% catechins +25% Duralox® and 50% catechins +50% Duralox® further suppress LOX activity, correlating with decreased HODE and off-flavors, while 250 ppm catechins and Duralox® treatments show intermediate inhibition, suggesting partial stabilization of hydroperoxides. However, the simultaneous rise in free radical levels and secondary oxidation products like hexanal suggests that non-enzymatic pathways, possibly promoted by redox-active components in Duralox® (*e.g.*, ascorbic acid), play a compensatory or even dominant role in downstream oxidation processes in Duralox® by alone. This indicates that antioxidants, particularly at higher balanced catechins levels in mixtures can also effectively mitigate LOX-mediated oxidation, reducing HODE accumulation and volatile off-flavor formation compared to the untreated control.

#### 3.2.5 Multivariate statistical analysis

Multivariate statistical analysis was performed on the results from the volatile organic compounds analysis to better distinguish and highlight the flavor differences among the different antioxidants and concentrations.

**Fig. 7:**
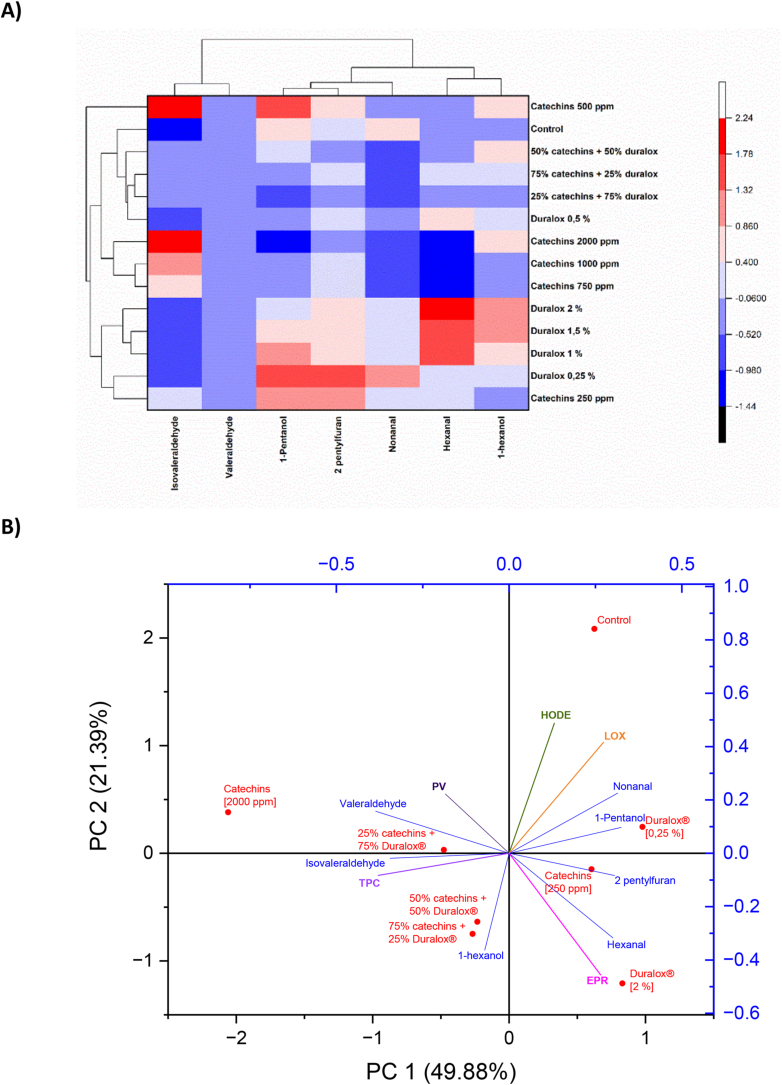
Clustered heatmap (7A) and PCA Biplot (7B) in volatile compounds analyzed via GC-MS of pea protein extracted with catechins, Duralox® and their mixtures. PV = peroxide value; HODE = hydroxyoctadecadienoic acids; LOX = lipoxygenase; EPR = electron paramagnetic resonance; TPC = total phenolic compounds.

The hierarchical clustering of volatile compounds in the treatments revealed two main groups that align closely with the dominant antioxidant present (Fig. 7A). An intermediate group included the control, catechins at 500 ppm, and 50% catechins:50% Duralox® mixture. The control, lacking antioxidants, produced a baseline oxidation profile representing a balance between hydroperoxide formation and degradation. The treatments displayed moderate suppression of certain volatiles but not to the extent observed in the catechins-dominant cluster. Catechins present in low concentration or balanced antioxidant ratios provided some protection but were insufficient to completely suppress volatile formation, placing these treatments between the two mechanistic extremes identified in the clustering.

Catechins-rich treatments, including 2000 ppm, 1000 ppm, and 750 ppm as well as the 75% catechins: 25% Duralox® mixture, clustered together. These samples showed strong suppression of most volatiles, particularly hexanal and nonanal. Alcohols such as 1-pentanol and 1-hexanol, along with furan derivatives, were also markedly reduced in these treatments. This volatile profile matches catechins' mode of action, characterized by strong LOX inhibition and effective quenching of free radicals.

A second major group comprised Duralox®-dominant treatments, including 2.0%, 1.5%, 1.0%, 0.5%, and 0.25% Duralox®, along with the 25% catechins:75% Duralox® mixture. These were clustered together because they retained higher levels of aldehydes and alcohols, particularly hexanal, nonanal, and 1-pentanol. This pattern indicates that while Duralox® partially inhibits lipid oxidation, it allows ongoing radical-mediated breakdown of hydroperoxides. This is consistent with the possibility of metal redox cycling promoted by Duralox® phenolics in the protein matrix, which can sustain radical production even when LOX activity is partially suppressed, as has been demonstrated in the results displayed in previous sections.

The clustering of volatile compounds further supports these interpretations. Hexanal and nonanal grouped together and were strongly suppressed in the catechins cluster. Isovaleraldehyde and valeraldehyde showed more variable behavior. In particular, valeraldehyde increased in some high-catechins treatments, suggesting enhanced protein oxidation at elevated catechins concentrations, as already explained. In Duralox®-rich treatments, 1-pentanol, 1-hexanol, 2-pentylfuran persisted, consistent with incomplete suppression of hydroperoxide breakdown. Together, the clustering patterns reveal that statistical similarity among treatments mirrors their underlying antioxidant mechanisms, with catechins achieving broad-spectrum suppression and Duralox® showing more selective and sometimes pro-oxidant effects.

These trends were further confirmed by PCA, which integrated volatile compounds (GC–MS data) with oxidative and antioxidant markers (Fig. 7B). In this analysis, peroxide value (PV), HODE, and electron paramagnetic resonance (EPR, representing free radicals) reflected the oxidative state of the system, while LOX represented an enzymatic driver of lipid oxidation. In contrast, TPC, introduced by catechins and Duralox®, contributed to oxidative stability by mitigating free radical formation and volatile production. The PCA biplot (PC1 = 49.88%, PC2 = 21.39%) accounted for 71.3% of the total variance.

The control sample was clearly separated on the positive side of PC1 and PC2, showing strong associations with volatiles such as nonanal and 1-pentanol, as well as with LOX and HODE. This indicates both a higher extent of lipid oxidation and limited suppression of LOX activity. This last result aligns with the LOX activity data (Fig. 6), where the control, lacking antioxidants, showed the highest enzyme activity.

Catechins at 2000 ppm was positioned on the negative side of PC1, closely related to TPC. Likewise, PV was also strongly associated with these high-phenolic samples, reflecting catechins' ability to scavenge peroxyl radicals, consistent with the results in Fig. 4B. Valeraldehyde was the only volatile strongly linked to high catechins content, which, as noted earlier, likely reflects protein rather than lipid oxidation. By contrast, catechins at 250 ppm clustered closer to 2-pentylfuran and hexanal, together with free radical levels, indicating an insufficient antioxidant effect. Given that 2-pentylfuran can also arise from singlet-oxygen–mediated oxidation of linoleic acid (L. Zhang et al., 2025), its presence may indicate that a portion of the lipid oxidation originated from non-enzymatic photooxidation. This raises the possibility that the antioxidants differed not only in their ability to limit hydroperoxide formation and decomposition, but also in their capacity to quench singlet oxygen. In this context, treatments containing catechins might be expected to mitigate this pathway more effectively than Duralox®, potentially explaining the relatively higher persistence of 2-pentylfuran in the latter.

Duralox® treatments displayed a concentration-dependent distribution. At 0.25%, samples were positioned near oxidation products such as nonanal and 1-pentanol, whereas higher concentrations were associated with elevated free radical signals (Fig. 5). Mixed treatments (catechins + Duralox®) clustered in the lower left quadrant, associated with PV and valeraldehyde, reflecting intermediate behavior and only partial reduction of oxidation products compared with single treatments.

Overall, PCA indicated that high TPC, particularly from catechins supplementation at 2000 ppm, was strongly associated with reduced lipid oxidation, suppression of LOX activity, and lower free radical levels.

### 3.3 Effect of antioxidant addition on sensory properties of pea protein

Sensory trials directly assess the perception, capturing nuanced attributes such as taste, aroma, flavor, appearance, and mouthfeel/texture, which chemical methods, *e.g.*, measuring HODE, aldehydes, volatiles, free radicals, LOX activity, cannot fully encapsulate. While chemical approaches provide precise data on the chemical suppression of off-flavor precursors, sensory evaluation translates these molecular changes into practical human perceptions. This is particularly crucial for pea protein isolates, where off-flavors, such as beany, rancid, or grassy notes, can hinder consumer acceptance, even when chemical profiles indicate reduced oxidation. Hence, incorporation of sensory data added a comprehensive understanding of how catechins and Duralox®, chosen for their effectiveness against volatile formation, affect the sensory quality of pea proteins, and that the observed enhancements can also be detected by human beings, ensuring alignment with consumer preferences and market demands.

**Fig. 8:**
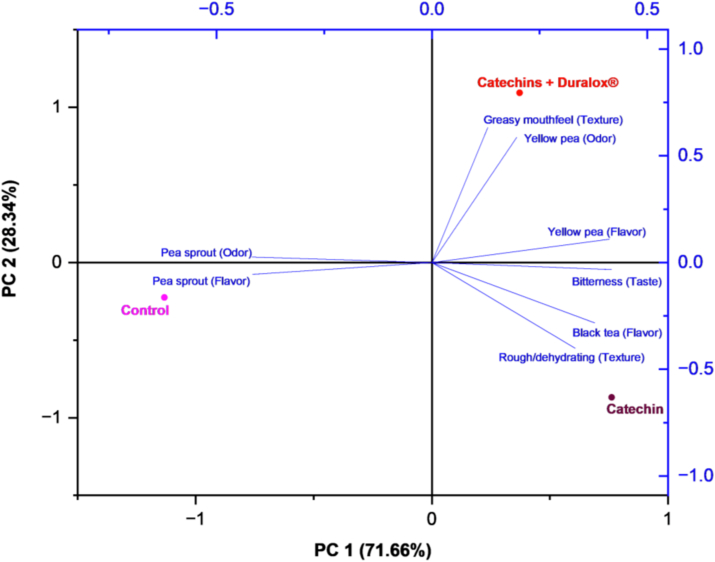
Principal component analysis (PCA) of the average sensory scores of 5% protein in water.

The PCA analysis of the average scores explained 100 % of the total data variation (Fig. 8). This indicates that the PCA model sufficiently captured the dominant differences among the treatments and sensory attributes. The data is also clustered in three main groups: control, catechins by alone and catechins + Duralox®, corresponding to one cluster each.

The control sample, which received no antioxidant treatment, was clearly separated from the antioxidant-treated samples along PC1, occupying the negative region. It aligned closely with sensory descriptors such as pea sprout (flavor) and pea sprout (odor), reflecting a strong expression of native green and vegetal notes characteristic of untreated pea proteins. These attributes are commonly associated with lipid-derived volatile compounds. More specifically, it has been stated that nine compounds, mainly aldehydes, are responsible for these pea-like sensory attributes, including 3-methylbutanal, hexanal, acetaldehyde, (E,E)-2,4-nonadienal, (E)-2-octenal, benzaldehyde, heptanal, 2-methylbutanal, and nonanoic acid (Utz et al., 2022). At the same time, as explained above, catechins inclusion reduced hexanal concentration from 6 to 1.2 μg g^−1^ DW, the primary aldehyde formed from linoleic acid oxidation and a key driver of beany, grassy, and green off-flavors due to its low odor threshold (Trigo et al., 2024). The close association of the control sample with pea sprout notes in the PCA therefore reflects the combined presence of these volatiles: alcohols and furans contributing to positive pea identity, alongside elevated hexanal intensifying the negative green and beany attributes. Grassy/green experience refers to sensory perception characterized by green or herbaceous notes, often caused by compounds like chlorophyll, aldehydes, and alcohols. While not a literal texture, it can involve slight astringency or dryness and is commonly associated with off-flavors such as "green," "beany," or "earthy." This sensation can detract from the overall eating experience and reduce consumer acceptance, especially in products where a neutral or creamy profile is expected (Leonard et al., 2023).

In contrast, the antioxidant-treated samples, particularly those treated with catechins alone or in combination with Duralox®, were positioned on the positive side of PC1, indicating a marked shift in sensory profile. This spatial separation suggests that catechins, when applied alone, effectively suppressed the perception of undesirable green and beany flavors, supporting its functionality as a lipid oxidation inhibitor during extraction. This positive sensory effect is consistent with the results in this study, for catechins which effectively mitigated different oxidation pathways and reduced the formation of various volatile compounds especially hexanal, which has a very low odor threshold value (Trigo et al., 2024). However, the catechins-treated samples (color brown in Fig. 8) clustered closely with descriptors such as bitterness, black tea flavor, and rough/dehydrating (*i.e.* astringency) mouthfeel. This suggests that while catechins are potent antioxidants, their polyphenolic structure introduces secondary sensory attributes which need further optimization. The emerged sensory attributes, such as black tea flavor, bitter taste, and astringent texture, are more likely attributable to the catechins themselves rather than to oxidation products, in line with previous studies (Y. Gao et al., 2025; Xiao et al., 2022). This interpretation is consistent with previous findings showing that catechins addition to lactose-reduced milk increased its bitterness and astringency, despite a marked inhibition of volatile compounds formation (Jansson et al., 2019). Mechanistically, catechins interact with salivary proteins via hydrogen bonding, leading to protein precipitation and an increase in oral friction due to the depletion of lubricating components. This enhances the perception of astringency (Rossetti et al., 2009). Moreover, catechins have been shown to activate transient receptor potential channels, specifically TRPA1 and TRPV1, on sensory neurons, contributing further to the astringent and irritant sensations in the oral cavity (Kurogi et al., 2012).

The catechins + Duralox® formulation showed a partial shift away from the harsher sensory axes, specifically bitterness and black tea notes, though it remains near yellow pea flavor/odor vectors. This intermediate positioning indicates modest improvements in sensory masking, suggesting a tempered release or perception of polyphenol addition effect, while preserving antioxidant efficacy. This indicates a more complex shift in sensory profile, where the combination treatment not only suppressed oxidation-related green notes but also retained or even enhanced core legume characteristics intrinsic to yellow pea. Notably, while yellow pea flavor and odor are less objectionable than green or beany volatiles, they still reflect the legume's identity. The proximity of the catechins + Duralox® treatment to these descriptors suggests that this combination may offer a balanced approach, mitigating undesirable oxidation off-flavors while preserving or gently modifying the inherent sensory qualities of the pea protein (Wu et al., 2021).

Overall, the findings demonstrate that antioxidant treatments, particularly catechins, are effective in reducing some sensory attributes associated with lipid oxidation. Catechins alone were highly effective in suppressing beany and green off-flavors compared to the control, but introduced new sensory challenges, including increased bitterness and astringency. Meanwhile, the combination of catechins and Duralox® appeared to provide a more moderate sensory shift, maintaining some desirable pea-related flavor attributes while still reducing oxidation-derived volatiles. These results underscore the importance of selecting and optimizing antioxidant treatments during the protein extraction, not only for oxidative stability but also for their broader impact on sensory quality. Depending on the intended food application, catechins may be particularly suitable when strong off-flavor masking is needed, while combination treatments may offer a more balanced sensory profile.

## 4 Conclusions

This study evaluated the effectiveness of selected antioxidants in inhibiting the formation or up-concentration of volatile off-flavor compounds during the extraction of pea protein using alkaline solubilization and isoelectric precipitation. The substantial formation of HODE during protein extraction demonstrated that lipid oxidation induced during the wet fractionation is the origin of the accumulation of the majority of undesirable volatile compounds. Screening various antioxidants revealed that the lipid oxidation during the protein extraction can be mitigated through antioxidant incorporation, where catechins and Duralox® were notably more effective. Both antioxidants significantly reduced the formation of lipid oxidation products (HODE and some volatile compounds). Further investigations into concentration effects and combinations of the two antioxidants revealed a dose-dependent response for catechins. Increasing its concentration from 250 ppm to 2000 ppm nearly eliminated the formation of several key off-flavor volatiles, including hexanal, hexanol and nonanal. In contrast, although Duralox® effectively suppressed primary lipid oxidation, increasing its concentration unexpectedly led to the accumulation of free radicals (as measured by EPR) and an increase in target off-flavor volatiles.

Catechins at an optimum concentration exhibited a strong inhibitory effect on lipid oxidation during the protein extraction, as evidenced by significantly lower levels of HODE, diminished hexanal formation in line with the substantial reduction in LOX activity (down to one-tenth of the control), and a significantly decreased EPR-detected free radicals. These findings suggest that catechins suppress both the enzymatic and non-enzymatic oxidation pathways. The consistent suppression across all oxidative markers highlights catechins' dual-mode functionality: inhibiting the enzymatic initiation of lipid oxidation while also directly quenching radicals and chelating pro-oxidant. Compared to Duralox®, which partially inhibited LOX yet promoted free radical accumulation and hexanal formation, catechins demonstrated a more comprehensive and dominant antioxidant mechanism under the wet fractionation conditions. However, increasing catechins concentration to above 500 ppm promoted the formation of valeraldehydes, often associated with protein oxidation.

Sensory analysis demonstrated that inhibiting lipid oxidation with antioxidant treatments during the protein extraction, particularly catechins, is effective in reducing some sensory attributes that may be considered unpleasant. Catechins alone were highly effective in suppressing beany (pea) and green off-flavors compared to the control. Nevertheless, catechins also introduced new sensory challenges, including increased bitterness and astringency. The combination of catechins and Duralox® offered a more balanced sensory profile but with less potent inhibition of off-flavor volatiles. Additionally, the side effect of catechins treatment on the color of the samples needs further investigation, which will be covered in our upcoming publications.

Overall, the findings underscore the potential of antioxidant addition during pea protein extraction to suppress lipid oxidation and associated off-flavor formation. However, the antioxidant type, dosage, and potential side effects on flavor, color, and protein quality need careful investigation and optimization to maximize benefits and minimize trade-offs.

## Declaration of competing interest

The authors declare that they have no known competing financial interests or personal relationships that could have appeared to influence the work reported in this paper.

## Data Availability

Data will be made available on request.
