# Supplementary material for: Screening and mechanistic evaluation of antioxidants for mitigating beany flavor formation during pea protein isolation
Source: Curr Res Food Sci. 2026 Apr 9;12:101400. doi: 10.1016/j.crfs.2026.101400 (PMC13122249; doi:10.1016/j.crfs.2026.101400)
Supplement: Multimedia component 1 [file mmc1.docx]

**Supplementary material SS1**. Volatile organic compounds in samples with different antioxidants

| Volatile compounds **[µg.g^-1^ DW]** | **Flour** | **Control** | **Catechins  (500 ppm - homog)** | **CSAE  (1% biomass)** | **Duralox  (1% biomass)** | **Duralox  (1% - homog)** | **CA  (100 ppm - homog)** |
| --- | --- | --- | --- | --- | --- | --- | --- |
| Isovaleraldehyde | 0.10 ± 0.00 | 0.12 ± 0.01 | 1.37 ± 0.10 | 0.11 ± 0.01 | 0.10 ± 0.00 | 0.23 ± 0.01 | 0.14 ± 0.02 |
| Valeraldehyde | 0.12 ± 0.00 | 0.18 ± 0.01 | 0.12 ± 0.02 | 0.23 ± 0.01 | 0.19 ± 0.03 | 0.17 ± 0.05 | 0.17 ± 0.01 |
| 3-methyl-1-butanol | 5.53 ± 0.53 | 4.12 ± 0.16 | 16.13 ± 1.14 | 7.42 ± 0.36 | 5.48 ± 0.07 | 8.94 ± 0.33 | 8.87 ± 0.67 |
| 1-pentanol | 7.16 ± 1.52 | 15.75 ± 1.34 | 4.59 ± 0.40 | 17.17 ± 0.92 | 15.90 ± 1.10 | 15.72 ± 0.85 | 15.82 ± 0.55 |
| Hexanal | 0.62 ± 0.18 | 3.60 ± 0.16 | 0.53 ± 0.06 | 3.09 ± 0.19 | 2.66 ± 0.21 | 3.59 ± 0.13 | 2.50 ± 0.55 |
| 1-Hexanol | 1.30 ± 0.13 | 1.56 ± 0.05 | 1.35 ± 0.01 | 2.74 ± 0.10 | 1.57 ± 0.34 | 1.33 ± 0.25 | 1.18 ± 0.21 |
| 2-pentylfuran | 0.01 ± 0.00 | 0.03 ± 0.00 | 0.01 ± 0.00 | 0.02 ± 0.00 | 0.01 ± 0.00 | 0.02 ± 0.00 | 0.02 ± 0.00 |
| 1-octen-3-ol | 0.33 ± 0.05 | 1.18 ± 0.07 | 0.94 ± 0.00 | 0.86 ± 0.04 | 0.47 ± 0.02 | 0.88 ± 0.01 | 0.78 ± 0.06 |
| 2-isopropyl-3-methoxy-pyrazine | 0.02 ± 0.00 | 0.03 ± 0.00 | 0.02 ± 0.00 | 0.01 ± 0.00 | 0.01 ± 0.00 | 0.01 ± 0.00 | 0.01 ± 0.00 |
| Nonanal | 0.01 ± 0.00 | 0.04 ± 0.00 | 0.01 ± 0.00 | 0.03 ± 0.00 | 0.02 ± 0.00 | 0.04 ± 0.00 | 0.03 ± 0.00 |
| 1-Nonanol | 0.03 ± 0.00 | 0.14 ± 0.01 | 0.08 ± 0.01 | 0.08 ± 0.00 | 0.07 ± 0.00 | 0.24 ± 0.03 | 0.14 ± 0.01 |
| E,E,2,4-nonadienal | 0.01 ± 0.00 | 0.01 ± 0.00 | 0.00 ± 0.00 | 0.01 ± 0.00 | 0.00 ± 0.00 | 0.02 ± 0.00 | 0.00 ± 0.00 |
| E,E,2,4-decadienal | 0.00 ± 0.00 | 0.00 ± 0.00 | 0.00 ± 0.00 | 0.00 ± 0.00 | 0.00 ± 0.00 | 0.00 ± 0.00 | 0.00 ± 0.00 |
